# Supplementary material for: A reconfigurable and magnetically responsive assembly for dynamic solar steam generation
Source: Nat Commun. 2022 Jul 27;13:4335. doi: 10.1038/s41467-022-32051-3 (PMC9329472; doi:10.1038/s41467-022-32051-3)
Supplement: Supplementary file 1 — Supplementary Information [file 41467_2022_32051_MOESM1_ESM.pdf]

Supplementary Information for

A reconfigurable and magnetically responsive assembly for dynamic solar steam generation

Yajie Hu[1], Hongyun Ma[1], Mingmao Wu[1], Tengyu Lin[1, 2], Houze Yao[1], Feng Liu[3] *, Huhu Cheng[1] *, and Liangti Qu[1] *

1 Key Laboratory of Organic Optoelectronics & Molecular Engineering, Ministry of Education, Department of Chemistry & State Key Laboratory of Tribology, Department of Mechanical Engineering, Tsinghua University, Beijing 100084, P. R. China

E-mail: huhucheng@tsinghua.edu.cn, lqu@mail.tsinghua.edu.cn

2 HurRain Nano Technology Co., Ltd, Beijing 100084, P. R. China

3 State Key Laboratory of Nonlinear Mechanics, Institute of Mechanics, Chinese Academy of Sciences, Beijing, 100190, P. R. China

E-mail: liufeng@imech.ac.cn

Supplementary Information

Contents

S1. Supplementary Notes

S2. Supplementary Figures

S3. Supplementary Tables

S4. Supplementary References

S1. Supplementary Notes

Supplementary Note 1. Derivation of the relationship between evaporation rate with surface temperature

For each point on the surface of the CA assembly, the heat inflow should be balanced with heat outflow, and as the solar thermal input for each point is the same, which can be described by,

$$Q_{in-sun} + Q_{in-envir} = Q_{evap}. \quad (1)$$

Q_{in-sun} refers to the heat induced by solar thermal effect which is the same for each point, $Q_{in-envir}$ refers to the heat flow from environment to the evaporator which should be positively correlated with $(T_{surr} - T)$, where T_{surr} is the temperature of surroundings and T is the surface temperature of point on the bevel of CA assembly , and Q_{evap} is the heat loss resulting from evaporation and should be equal to $\Delta H \times R$, where ΔH is the enthalpy of evaporation, and R is the evaporation rate. Thereafter, given that ΔH is nearly a constant in this temperature range, Supplementary equation (2) could be derived as follows:

$$R \propto (T_{surr} - T). \quad (2)$$

From this equation, it can be concluded that the cooler point has the higher evaporation rate owing to more heat inflow from the environment.

Supplementary Note 2. Derivation of the coupling holistic evaporation rate

At the bevel of the cone, the thermal conduction differential equation[1] can be expressed as,

$$-\kappa \cdot \nabla^2 T = Q_{in-sun} + Q_{evap}. \quad (3)$$

Among them, κ is the heat conductivity coefficient for the assembly and the damp air, corresponding to 0.59 $\mathrm{W} \cdot m^{-1} \cdot K^{-1}$ and 0.026 $\mathrm{W} \cdot m^{-1} \cdot K^{-1}$ respectively. Q_{in-sun} is the solar-thermal heat source. Because the solar-thermal power is $1000\ \mathrm{W} \cdot m^{-2}$ for the projected area, thus Q_{in-sun} for the bevel of the cone can be given by $Q_{in-sun} = 1000 \times \frac{2}{\sqrt{2^2+5^2}} \mathrm{W} \cdot m^{-2} = 371.39\ \mathrm{W} \cdot m^{-2}$. And Q_{evap} is the heat consumption by water evaporation, and its value can be given by $Q_{evap} = -\Delta H_{evap} \cdot g_{evap}$, ΔH_{evap} is the enthalpy of evaporation which equals $40.8\ \mathrm{kJ} \cdot mol^{-1}$, g_{evap} is the evaporation rate at the surface.

According to our proposed mechanism, the collective evaporation process can be divided into two consecutive processes: interfacial evaporation and water vapor diffusion. The interfacial evaporation rate could be described by Dalton's evaporation[2,3] equation1, given by

$$g_{evap} = K \cdot (p^* - p), \tag{4}$$

where p^* is the saturated vapor pressure, and p is the vapor pressure near over the surface, K is the evaporation coefficient, equals $2.88 \times 10^{-7} s \cdot m^{-1}$ here, which is obtained from the experiment. And water vapor diffusion rate could be described by Fick's first law, which is

$$\boldsymbol{g_w} = -D \cdot \nabla P, \tag{5}$$

where $\boldsymbol{g_w}$ refers to the diffusion flux of water vapor, D is the diffusion coefficient which is $2.6 \times 10^{-5} m^2 \cdot s^{-1}$ for 28 °C water vapor, P is the water vapor pressure in the whole space. For evaporation process in a continuous and steady state, we could make steady-state approximation, i.e., p remains constant and g_{evap} equals the

normal component of $\boldsymbol{g_w}$. Here we get

$$g_{evap} = \boldsymbol{n} \cdot \boldsymbol{g_w}, \tag{6}$$

where $\boldsymbol{n}$ is the normal unit vector. Therefore, the governing equations in this system could be given by

$$-\kappa \cdot \nabla^2 T = 371.39\ \mathrm{W} \cdot m^{-2} - \Delta H_{evap} \cdot g_{evap}, \tag{7}$$

$$g_{evap} = K \cdot (p^* - p) = -D \cdot \boldsymbol{n} \cdot \nabla P, \tag{8}$$

and can be numerically solved by finite element method. In detail, the model is set to a cone with bottom radius = 2 mm, height = 5 mm, which is surrounded by cubic shaped damp air with side length = 30 mm. The relative humidity and temperature of the air boundary is set to 50% and 28°C respectively.

Supplementary Note 3. Clarification for the relationship between stability of the macroscopic conic shape and the microscopic nanoparticles' rearrangement

As shown in Supplementary Figure 36, $<r^2>$ represents the mean square movement radius for the nanoparticles during rotation. For comparison, three situations were simulated including the rigid-body rotation, dynamic rotation with relaxation time of nanoparticles much shorter than that of the rotation and dynamic rotation with relaxation time of nanoparticles comparable to that of the rotation, which are labeled as rigid body, fast dynamic and slow dynamic rotation, respectively. It can be seen, for fast dynamic rotation, $<r^2>$ increases much faster than that of the rigid body, and reaches a stable value comparable to the highest level of the rigid body, indicating the rapid rearrangement of the nanoparticles in the CA assembly. However, for slow dynamic rotation, $<r^2>$ increases slowly, and reaches a much lower level than that of the stable

rotation. Accordingly, the macroscopic conic shape for the fast dynamic rotation remains stable while that of the slow dynamic rotation contorts a lot and loses its stability as a conic assembly (Supplementary Figure 37). At the same time, the trajectory of four individual nanoparticles in these two conditions confirms that nanoparticles get no enough time in slow dynamic rotation, resulting in much lower degree of reconfiguration, as well as worse stability of the conic shape (Supplementary Figure 38). In experiments, the conic shape is quite stable even under rotation rate of 200 rpm, indicating its good stability. Therefore, in the CA assembly, the nanoparticles should undergo a fast reconfiguration process with relaxation time much shorter than that of the macroscopic rotation, which also accounts for stability of the conic shape.

Supplementary Note 4. Calculation flow and program construction of the Monte Carlo simulation.

As shown in Supplementary Figure 35, according to the classic Monte Carlo method[4], probability determined evolution is conducted. Firstly, the initial configuration (including coordinates and the dipole moments of particles) is set to a pre-relaxed tilted cone consisting of 2000 particles (the stable configuration in the initial external magnetic field). In each step, every particle in the system undergoes a possible configuration change process. Specifically, for each particle, a Monte Carlo step length is passed to a random direction which will change the total energy of the whole configuration. By comparing the energy of the new and current configuration, a transition state condition is judged to determine whether this particle movement could happen or not. After every particle accomplishing this process, the program changes the

orientation of the external magnetic field and runs into the next Monte Carlo step until the end (step = MC_step).

S2. Supplementary Figures

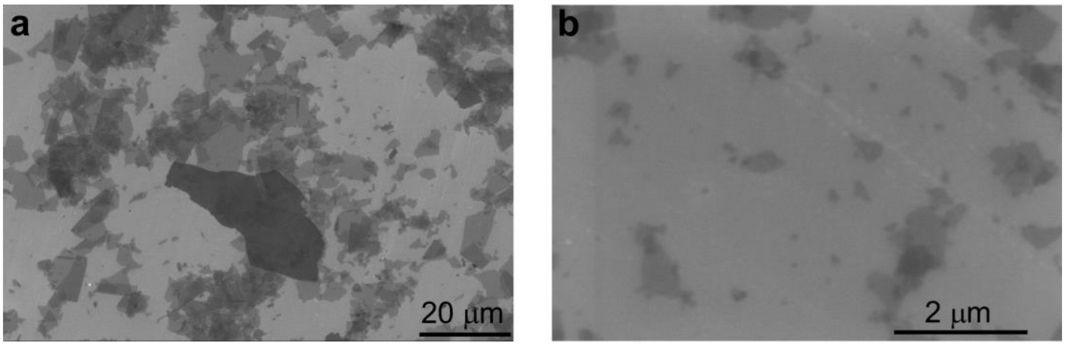

Supplementary Figure 1. SEM images of GO sheets (a), and GO sheets after ultrasonication (b).

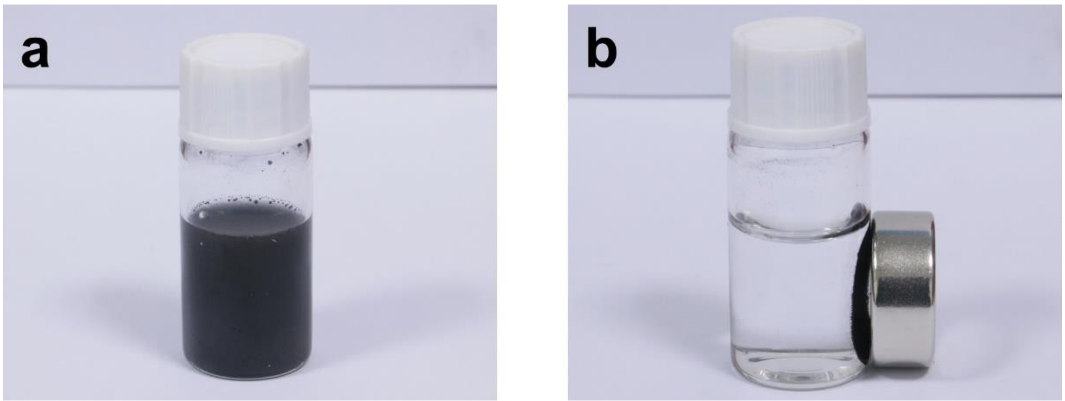

Supplementary Figure 2. Optical images of evenly dispersed Fe_3O_4@GO (a) and collection by magnet within 5 s (b).

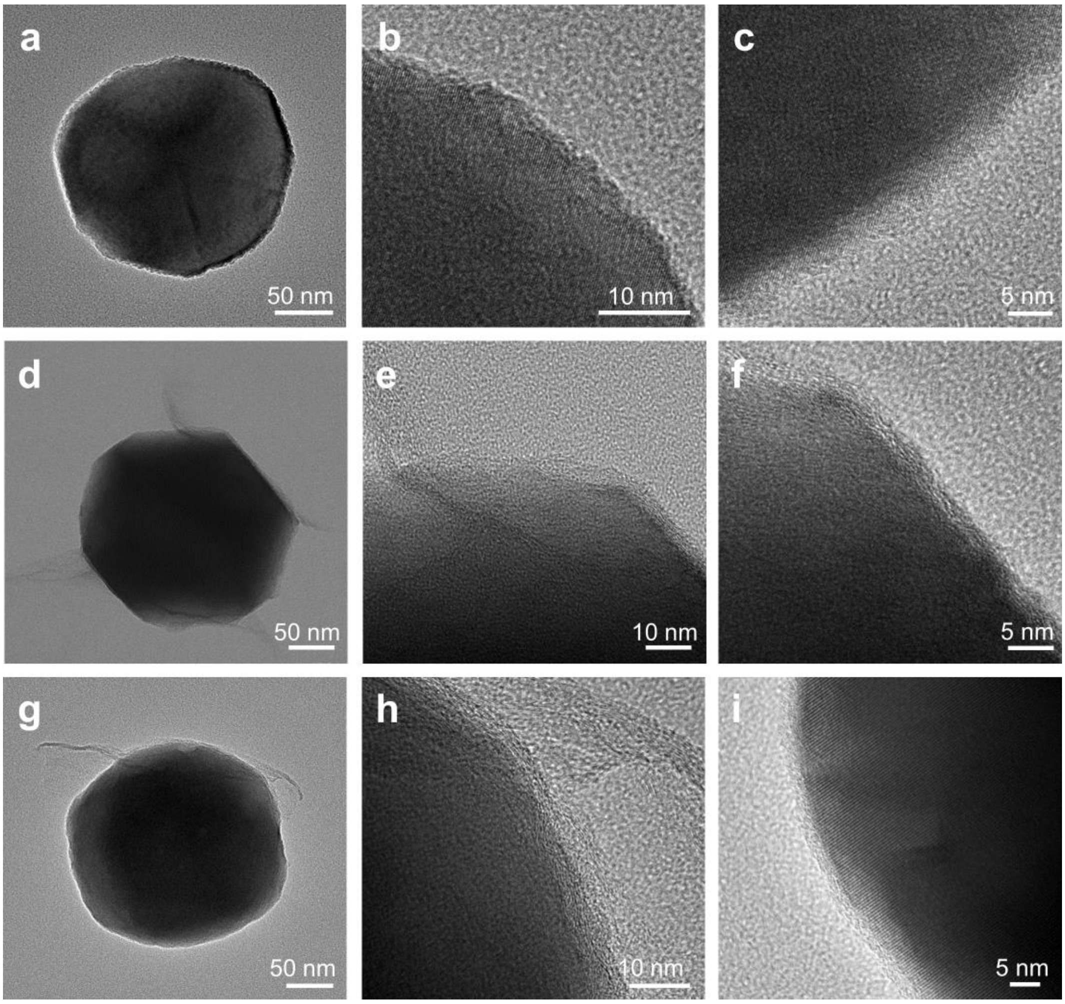

Supplementary Figure 3. TEM images of Bare Fe_3O_4 nanoparticle (a-c), Fe_3O_4@GO nanoparticle (d-f), and Fe_3O_4@G nanoparticle (g-i) at different magnified scale.

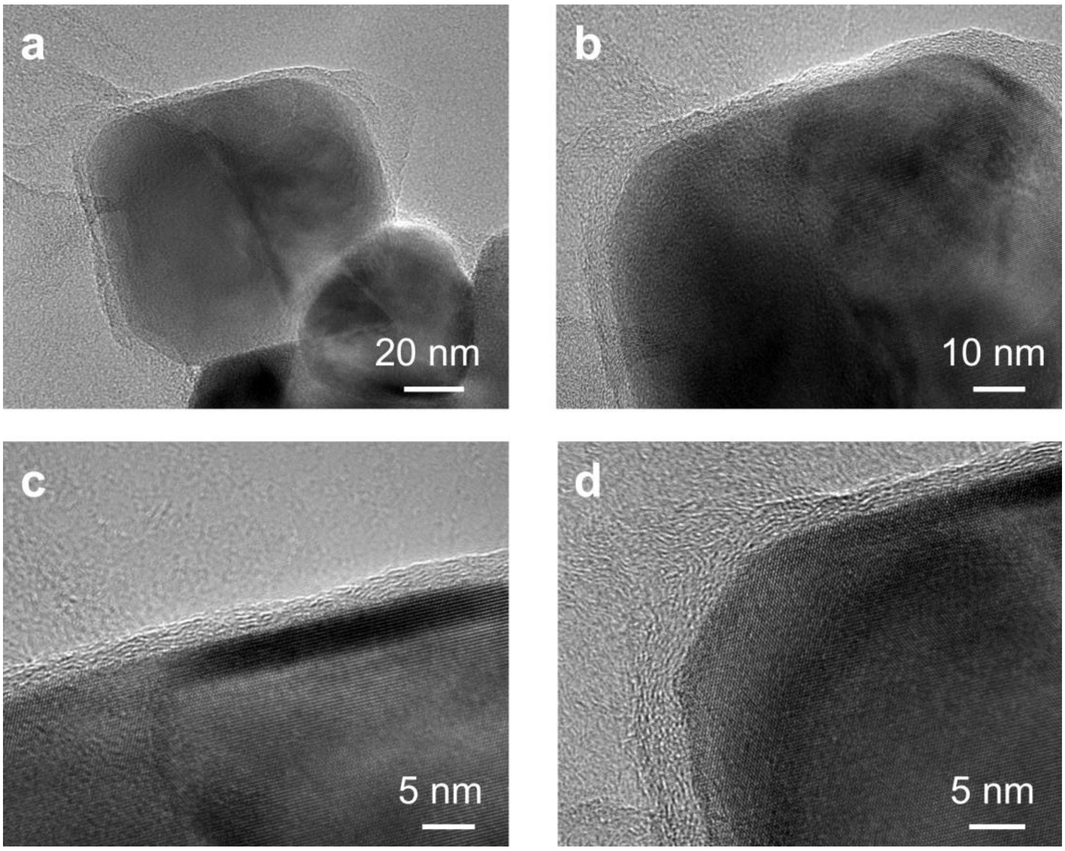

Supplementary Figure 4. HRTEM images of Fe_3O_4@G nanoparticle at different magnified scale.

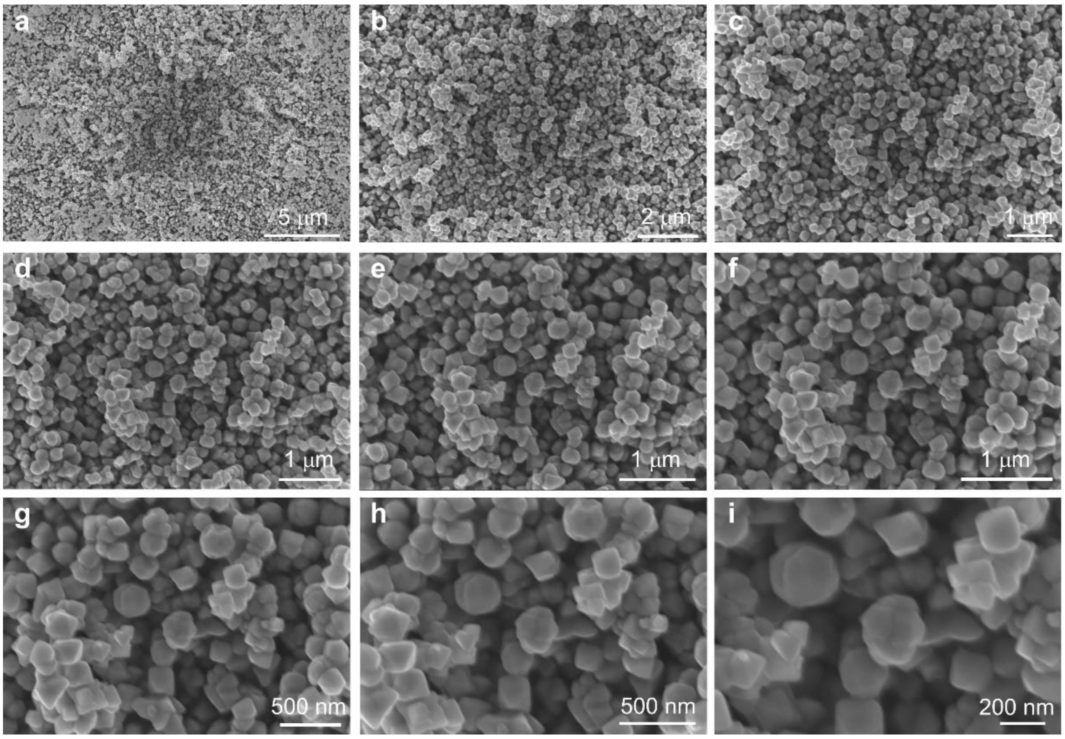

Supplementary Figure 5. SEM images of bare Fe_3O_4 nanoparticles at different

magnified scale.

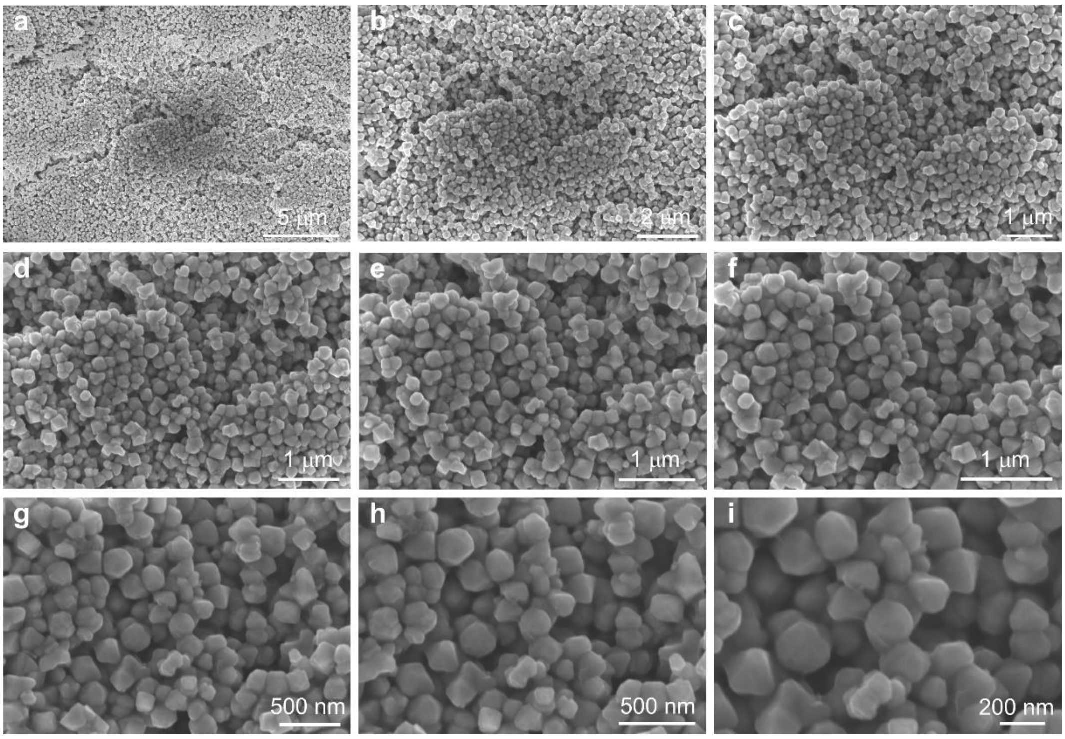

Supplementary Figure 6. SEM images of Fe_3O_4@GO nanoparticles at different magnified scale.

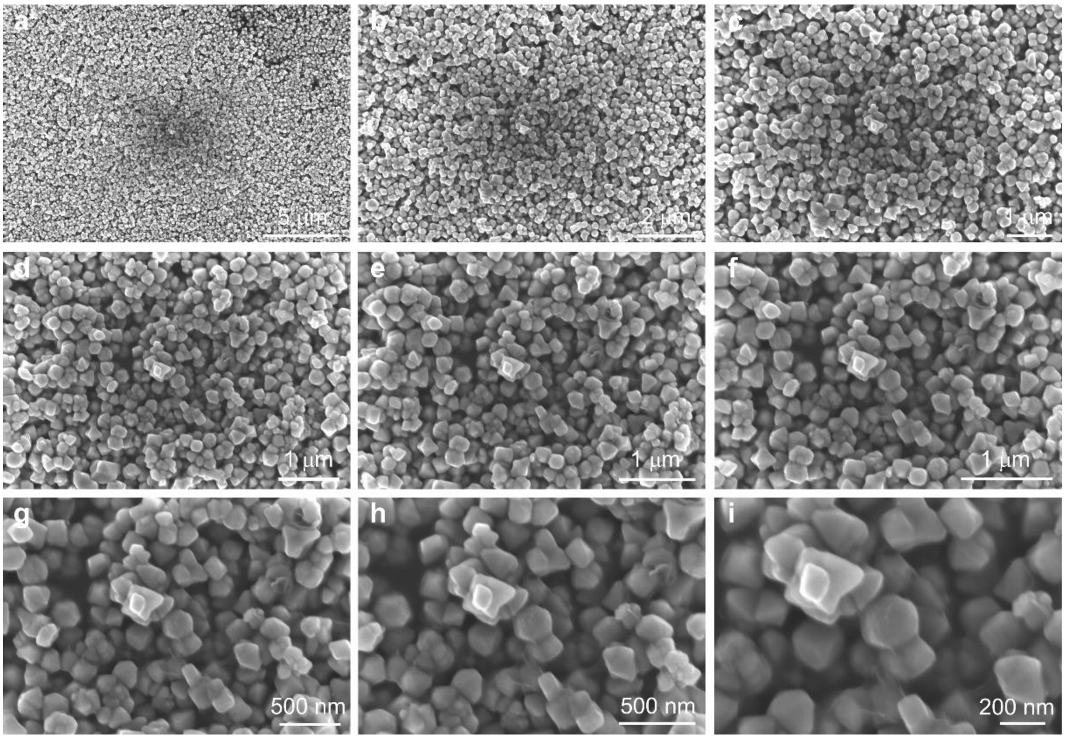

Supplementary Figure 7. SEM images of Fe_3O_4@G nanoparticles at different

magnified scale.

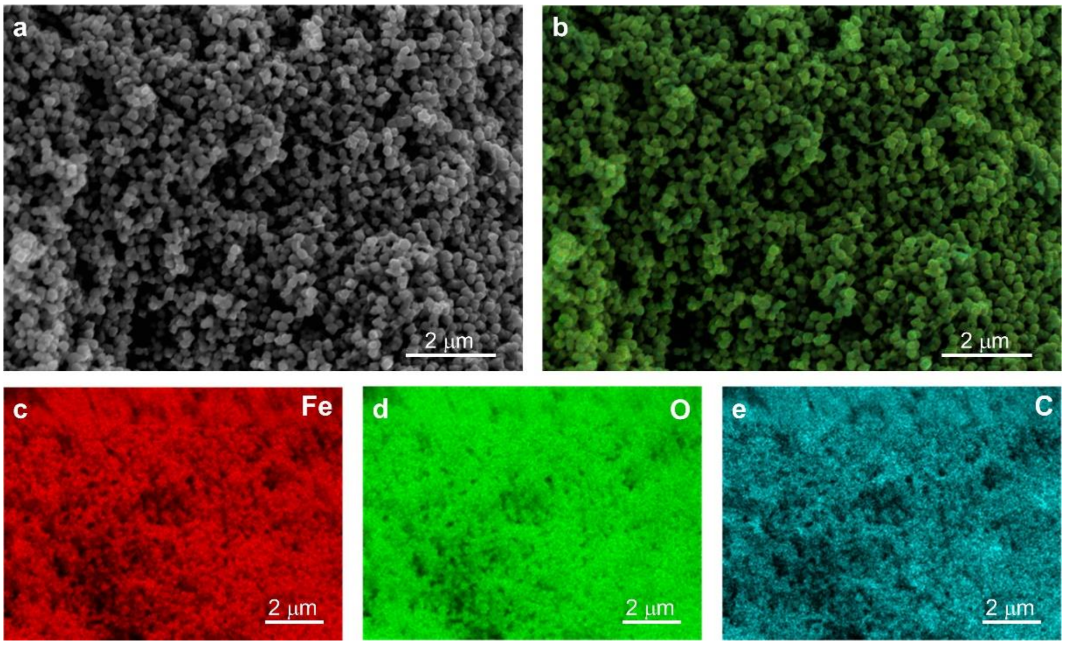

Supplementary Figure 8. EDS mapping of Fe_3O_4@G nanoparticles. The original SEM image (a), the composite image of different elements (b). EDS mappings of Fe (c), O (d), and C (e) elements. The uniformly distributed C element confirmed the graphene component in Fe_3O_4@G nanoparticles.

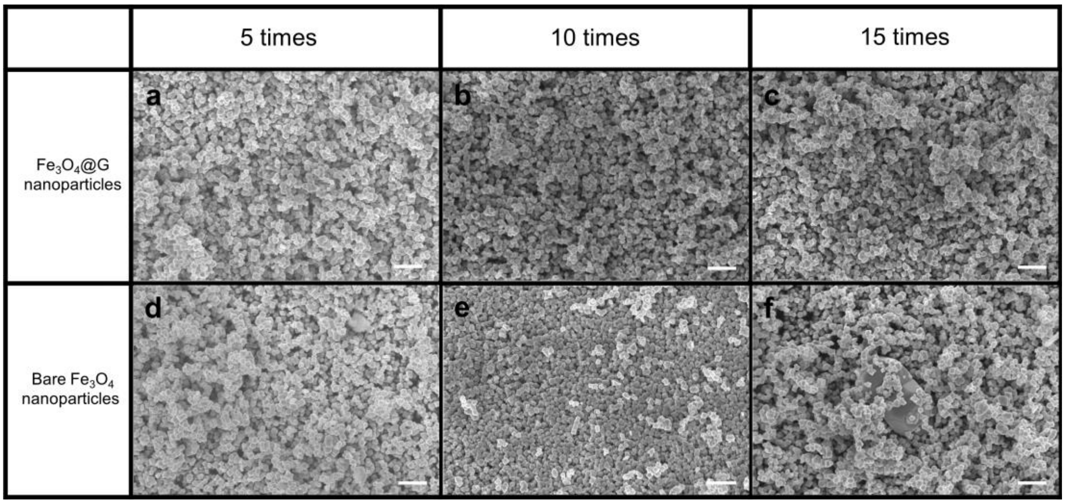

Supplementary Figure 9. SEM images of bare Fe_3O_4 nanoparticles and Fe_3O_4@G nanoparticles after recycling. Scale bar, 1 μm. (a-c) Uniformly distributed Fe_3O_4@G nanoparticles. No change was found after recycling. (d-f) Aggregation and large

particles in bare Fe_3O_4 nanoparticles as recycling times increasing.

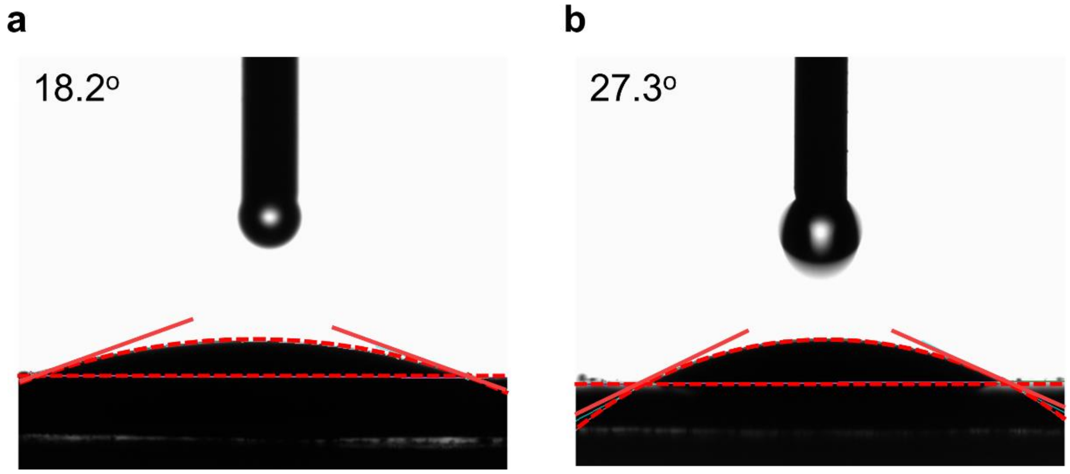

Supplementary Figure 10. Intrinsic contact angle of bare Fe_3O_4 (a) and Fe_3O_4@G nanoparticles (b).

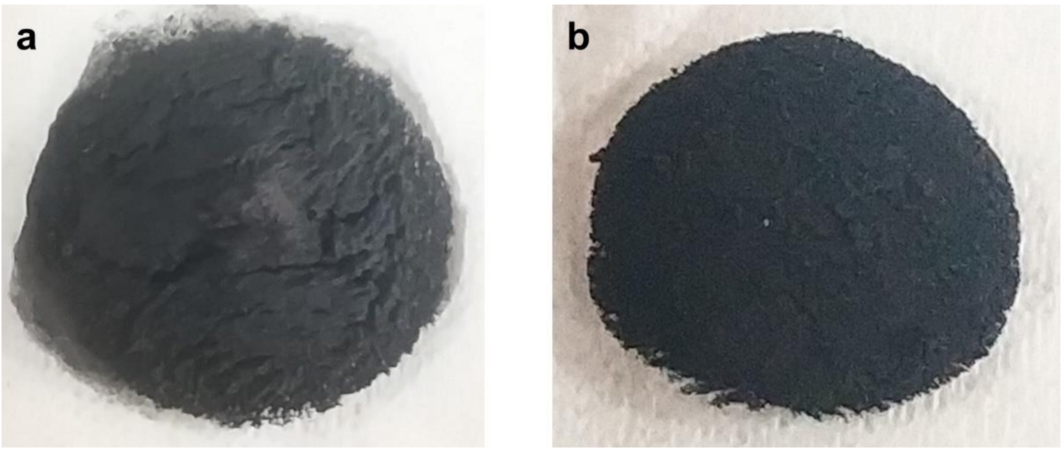

Supplementary Figure 11. Optical images of evaporation induced assemblies of bare Fe_3O_4 (a) and Fe_3O_4@G nanoparticles (b) at 60 °C. Large cracks were found in the assembly of bare Fe_3O_4 nanoparticles, resulting from the strong capillary force induced by evaporation, while a “smooth” surface was shown for the assembly of Fe_3O_4@G nanoparticles. Scale bar, 1 mm.

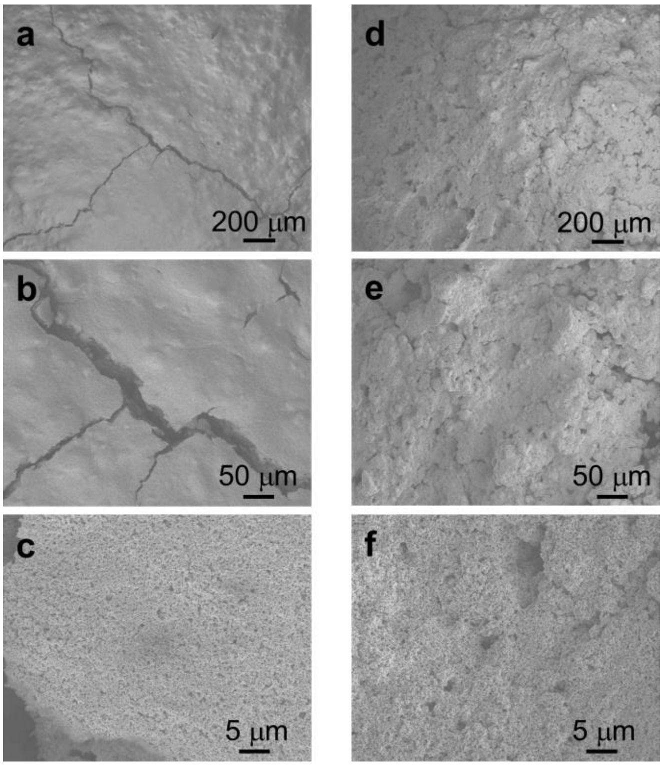

Supplementary Figure 12. SEM images of evaporation induced assemblies of bare Fe_3O_4 and Fe_3O_4@G nanoparticles at different magnified scale. (a-c) Huge cracks and compact aggregation were found for bare Fe_3O_4, (d-f) a continuous surface with multiple different sized pores was shown in assemblies of Fe_3O_4@G nanoparticles.

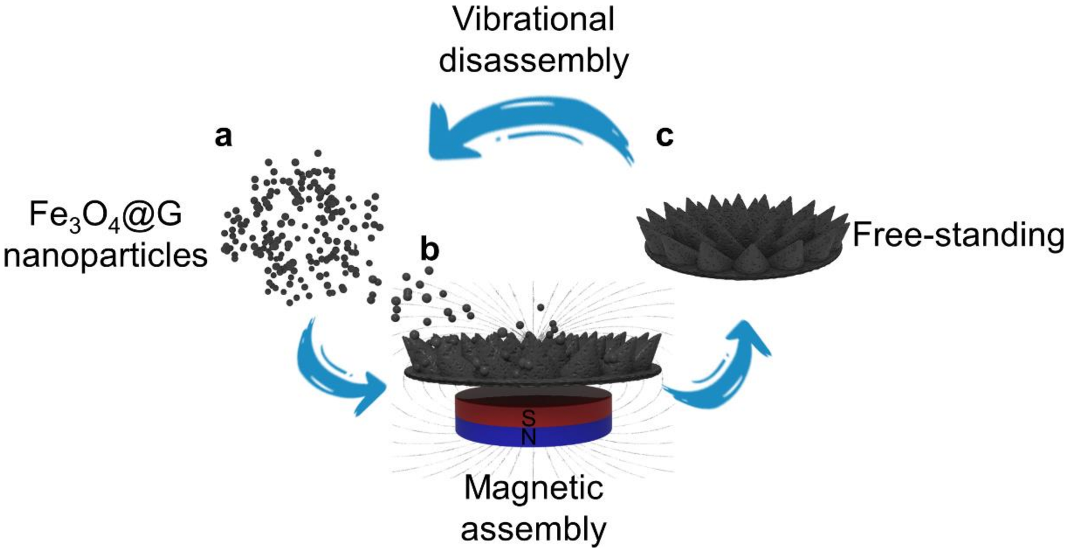

Supplementary Figure 13. Schematic image of the reconfiguration cycle of Fe_3O_4@G nanoparticles. (a) Discrete Fe_3O_4@G nanoparticles. (b) The magnetic

assembly process (c) The free-standing CA assembly.

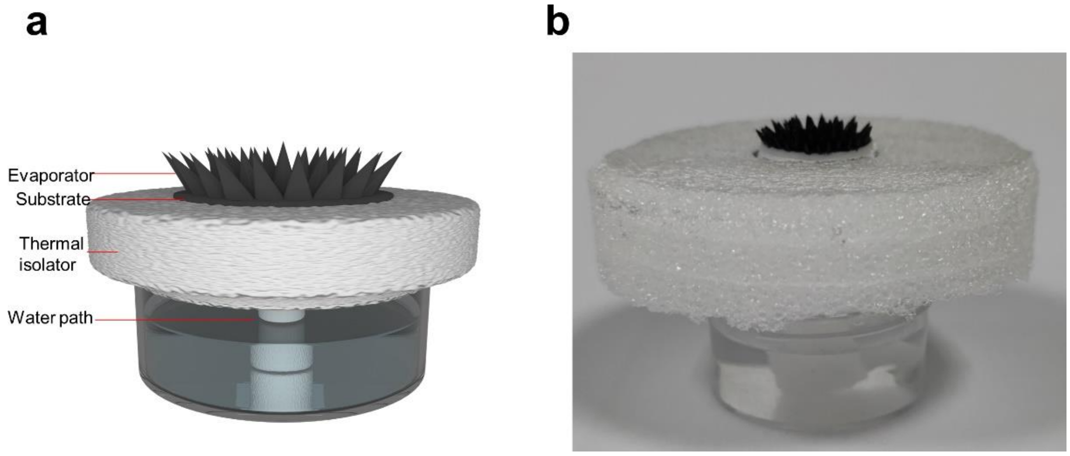

Supplementary Figure 14. (a) Schematic and (b) optical image of static evaporation apparatus.

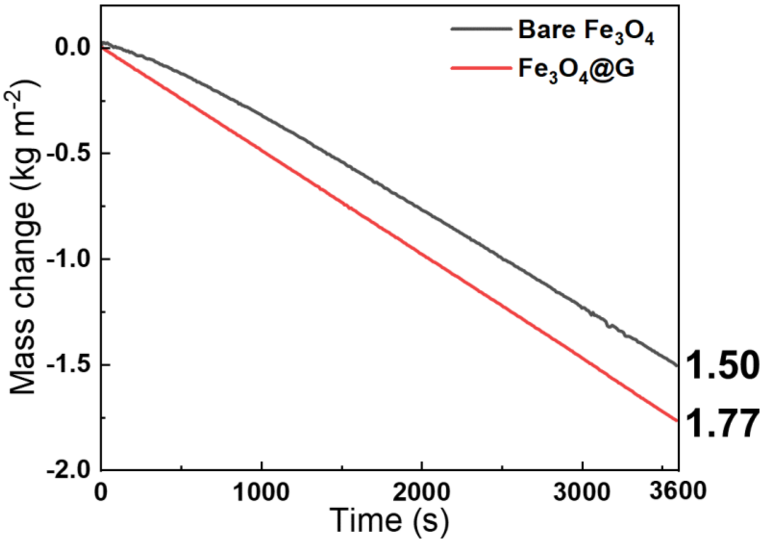

Supplementary Figure 15. Evaporation performance comparison between bare Fe_3O_4 and Fe_3O_4@G nanoparticle's CA assembly under 1 sun illumination. The evaporation rate of bare Fe_3O_4 nanoparticles' assembly is only 1.50 kg m^{-2} h^{-1}, while that of the Fe_3O_4@G nanoparticles' assembly is 1.77 kg m^{-2} h^{-1}, which is 18% higher.

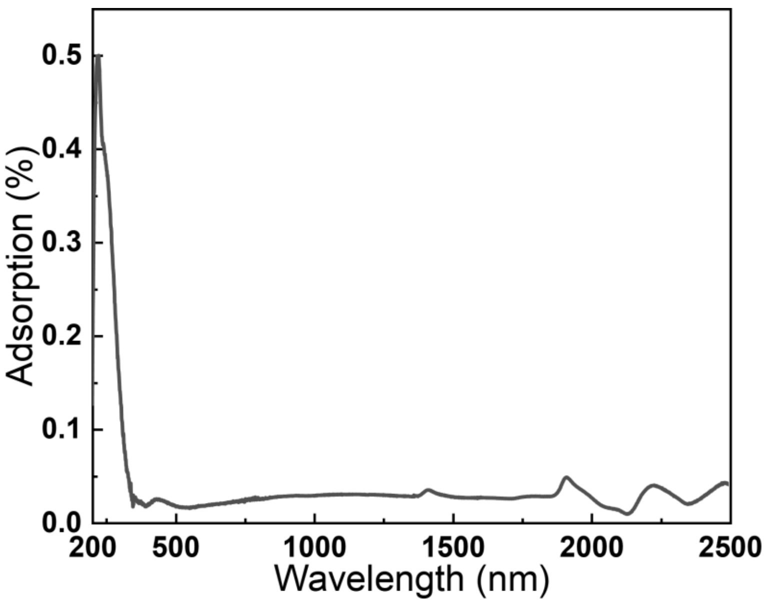

Supplementary Figure 16. UV-Vis-NIR absorption spectrum of glass fiber substrate.

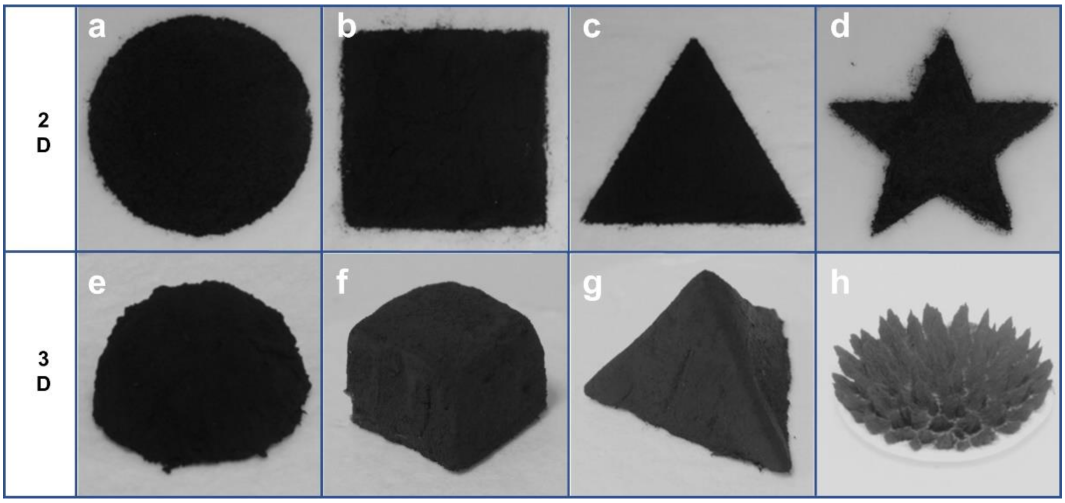

Supplementary Figure 17. 2D and 3D evaporators with different shapes constructed by Fe_3O_4@G nanoparticles, i.e., 2D shapes including plain (a), square (b), triangle (c), and star (d), 3D shapes including hemisphere (e), cube (f), pyramid (g), and CA assembly (h).

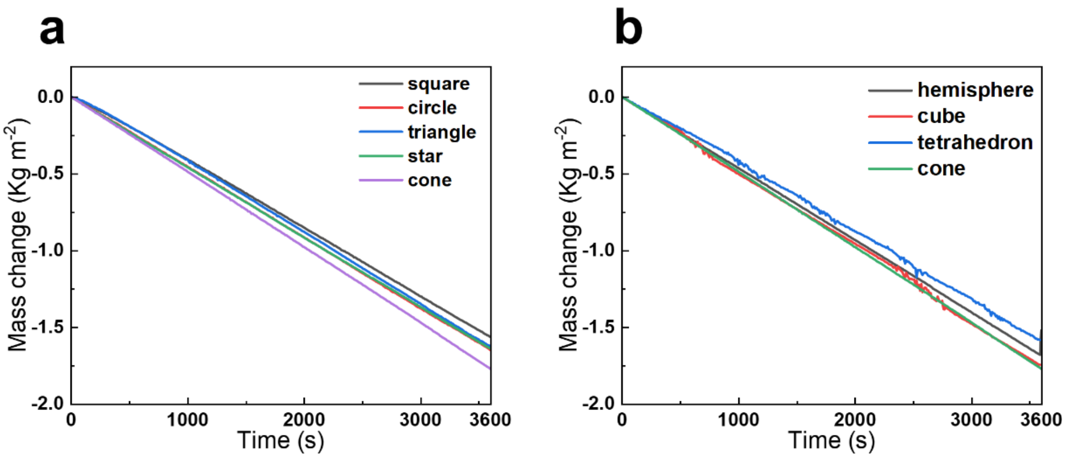

Supplementary Figure 18. Mass change of different 2D (a) and 3D (b) shaped Fe_3O_4@G assemblies under 1 sun illumination. Compared with 2D shapes, Fe_3O_4@G CA assembly owns the highest evaporation rate. Compared with 3D shapes, the evaporation rates of CA assembly and cube shaped assembly are equally the highest, while the mass of cube is 4.67g, which is about 18 times larger than that of the CA assembly.

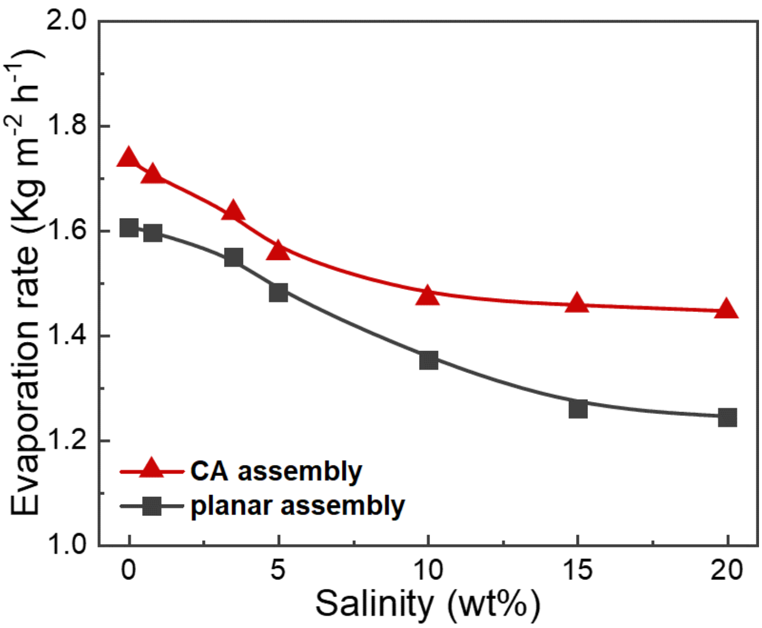

Supplementary Figure 19. Evaporation rate of CA vs. planar assemblies using simulated seawater with different salinity under 1 sun illumination.

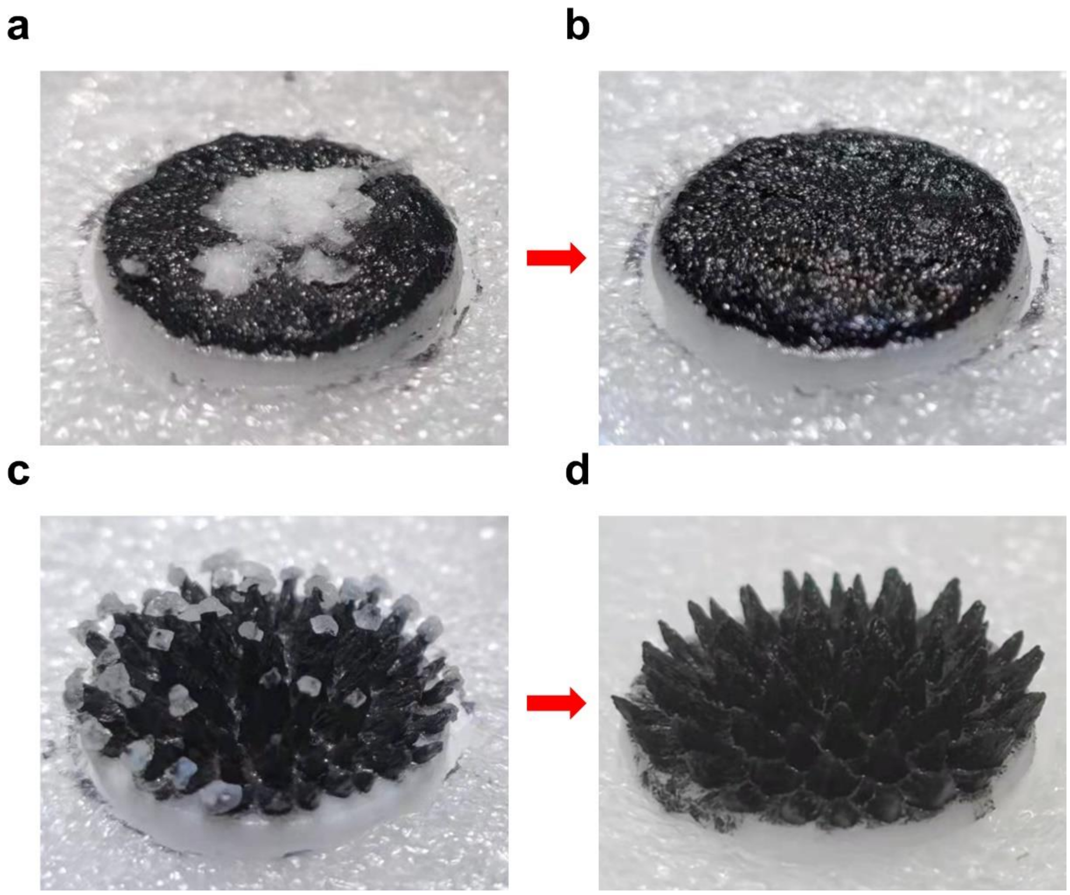

Supplementary Figure 20. Self-dissolution of precipitated salt on the evaporator. As illustrated, the precipitated salts were fully re-dissolved after several hours' quiescence, indicating the water transfer in the assembly are highly unimpeded.

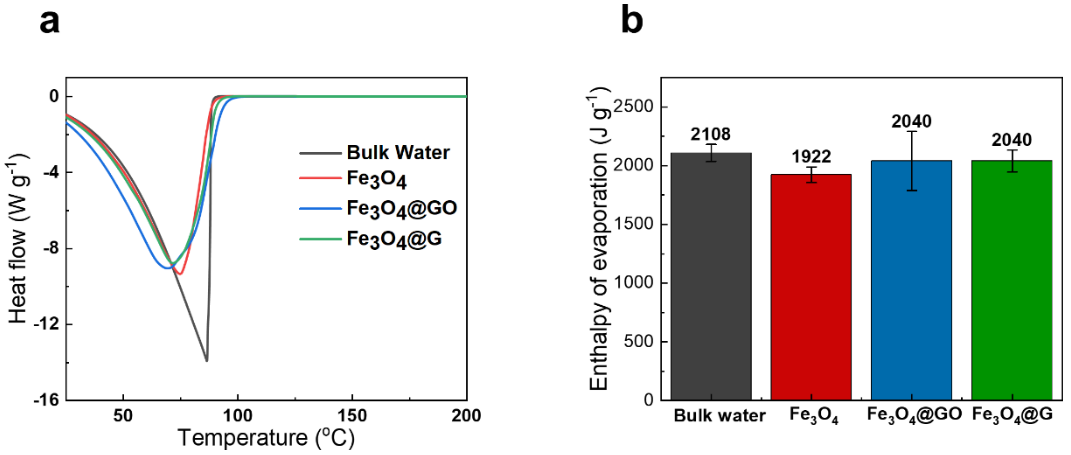

Supplementary Figure 21. (a) DSC spectrum and (b) calculated enthalpy of evaporation of bulk water and water contained in different nanoparticles (n = 3, error bars: standard deviation).

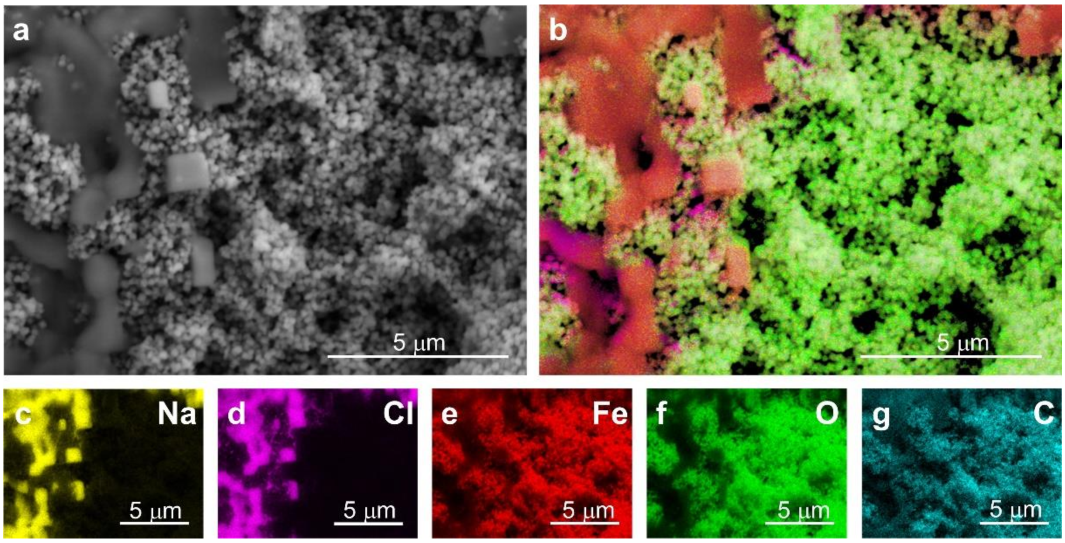

Supplementary Figure 22. EDS mapping of slat-fueled Fe_3O_4@G nanoparticles. (a) The original SEM image, (b) the composite image of different elements, EDS mappings of Na (c), Cl (d), Fe (e), O (f), and C (g) elements. It can be seen that salts tend not to precipitate together with Fe_3O_4@G nanoparticles, becoming discrete portions eventually.

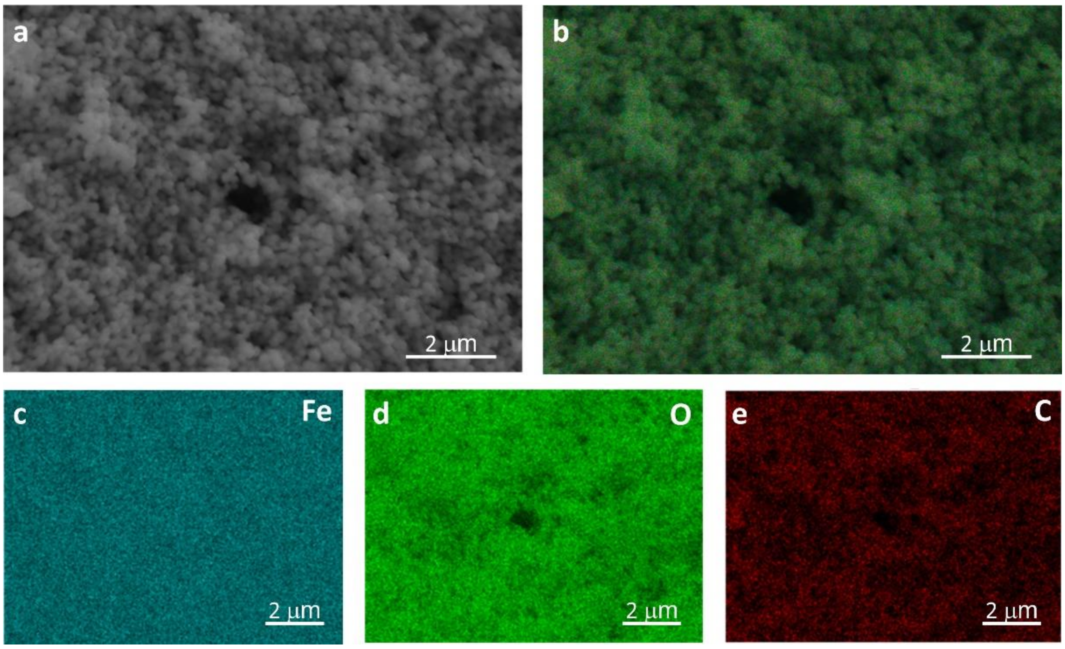

Supplementary Figure 23. EDS mapping of Fe_3O_4@G nanoparticles after 10 times' regeneration. (a) The original SEM image, (b) the composite image of different elements, EDS mappings of Fe (c), O (d), and C (e)elements. Even after 10 times'

regeneration, there was no trace of salt precipitation, which insured the persisting high evaporation rate.

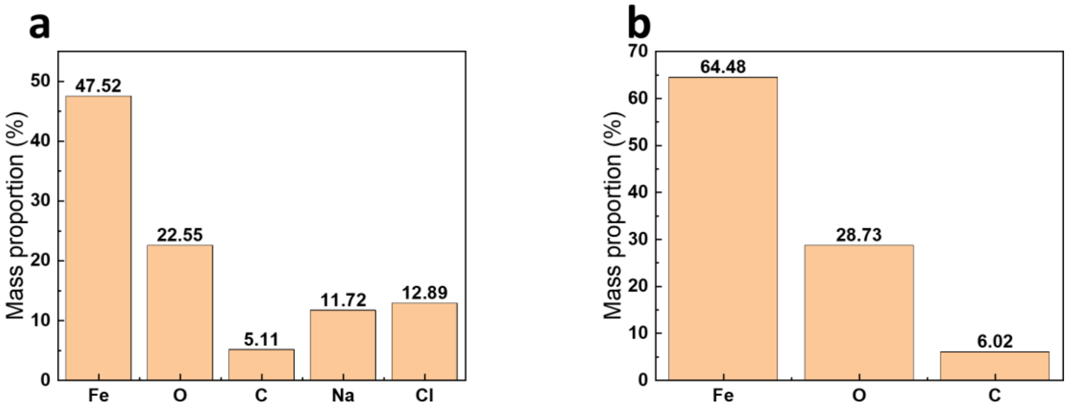

Supplementary Figure 24. Element proportion of slat-fueled (a) and recovered (b) Fe_3O_4@G nanoparticles after 10 times' regeneration from EDS.

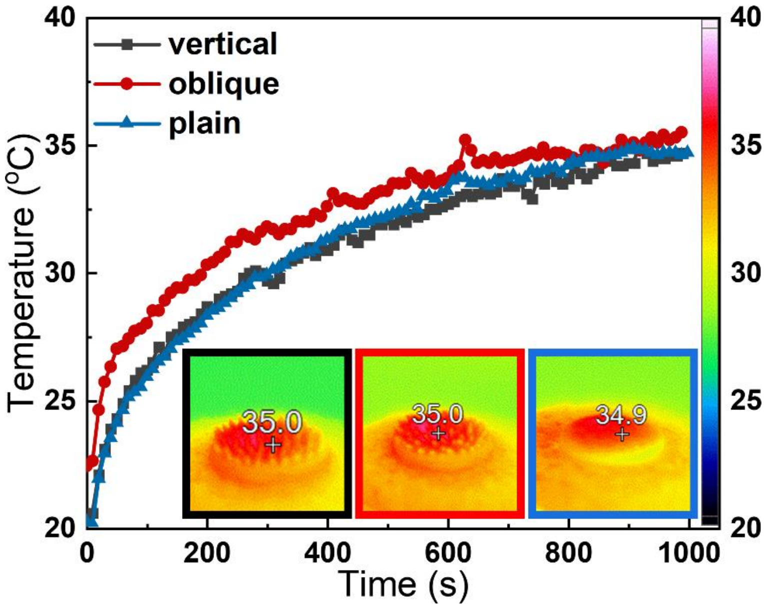

Supplementary Figure 25. Infrared images of vertical, oblique CA assembly, and plain shaped Fe_3O_4@G assemblies under 1 sun illumination. There is nearly no difference of sunlight absorption for the three shaped assemblies.

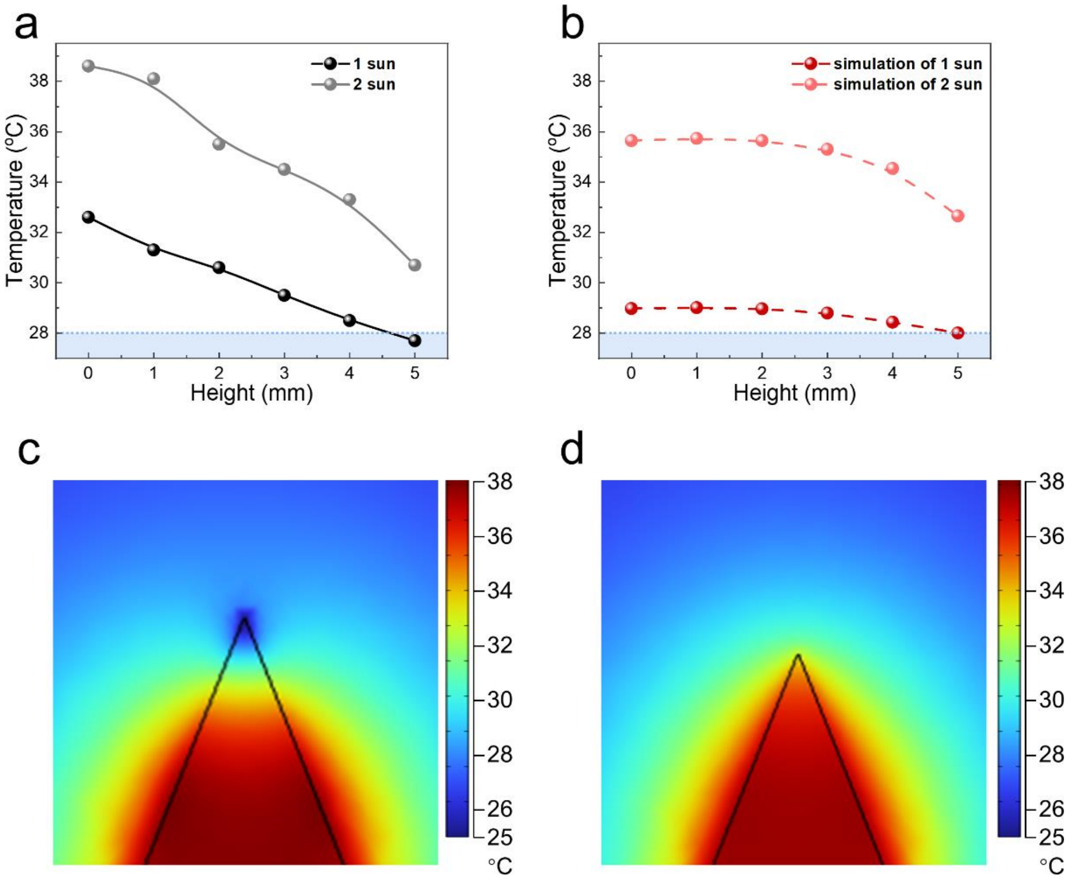

Supplementary Figure 26. Numerical calculation results of the temperature distribution of the CA assembly under 1 and 2 sun illumination. (a) The experimental results and (b) the simulated results. (c, d) The simulated temperature distribution diagrams of the cone under 1 sun (c) and 2 sun (d) illumination.

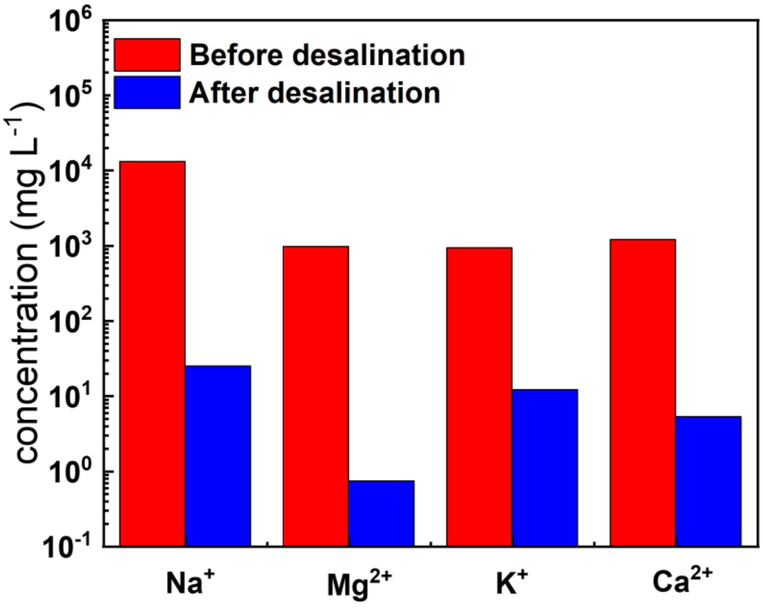

Supplementary Figure 27. Concentration of salt ions before and after desalination.

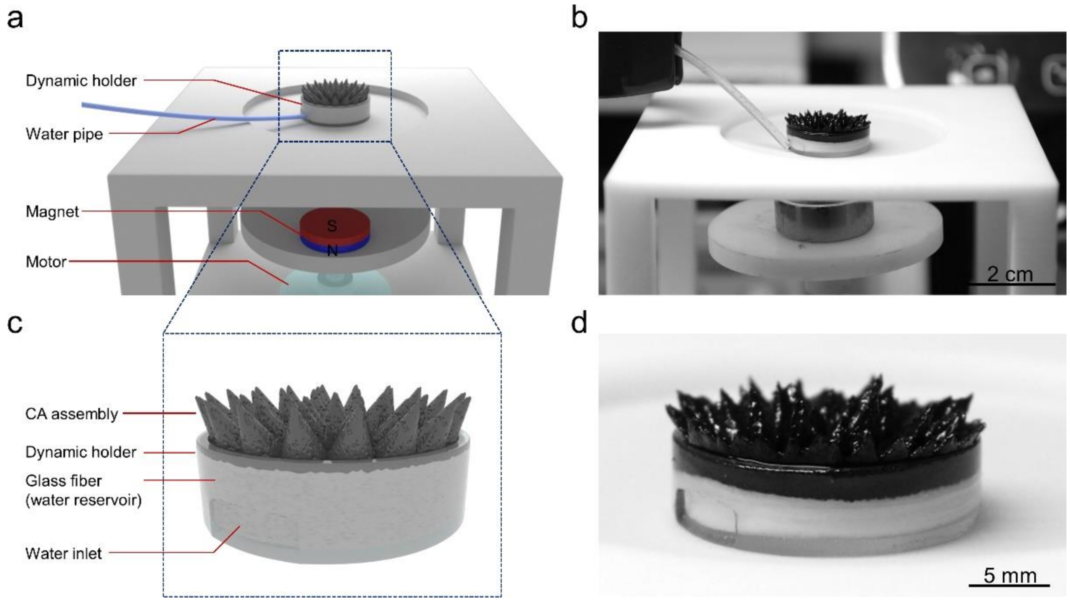

Supplementary Figure 28. Schematic and optical images of the dynamic evaporation apparatus. (a, b) The schematic image (a) and optical image (b) of the dynamic apparatus. (c, d) The schematic image (c) and the optical image (d) of the dynamic holder.

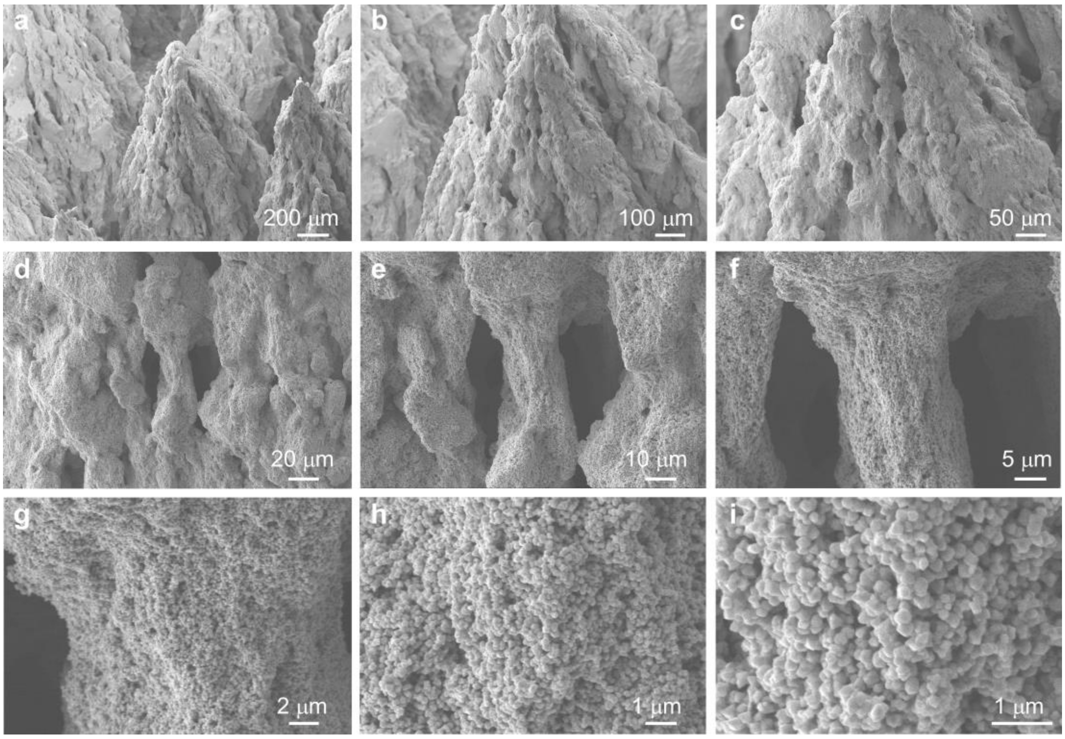

Supplementary Figure 29. SEM images of the vertical conic assembly at different

magnified scale.

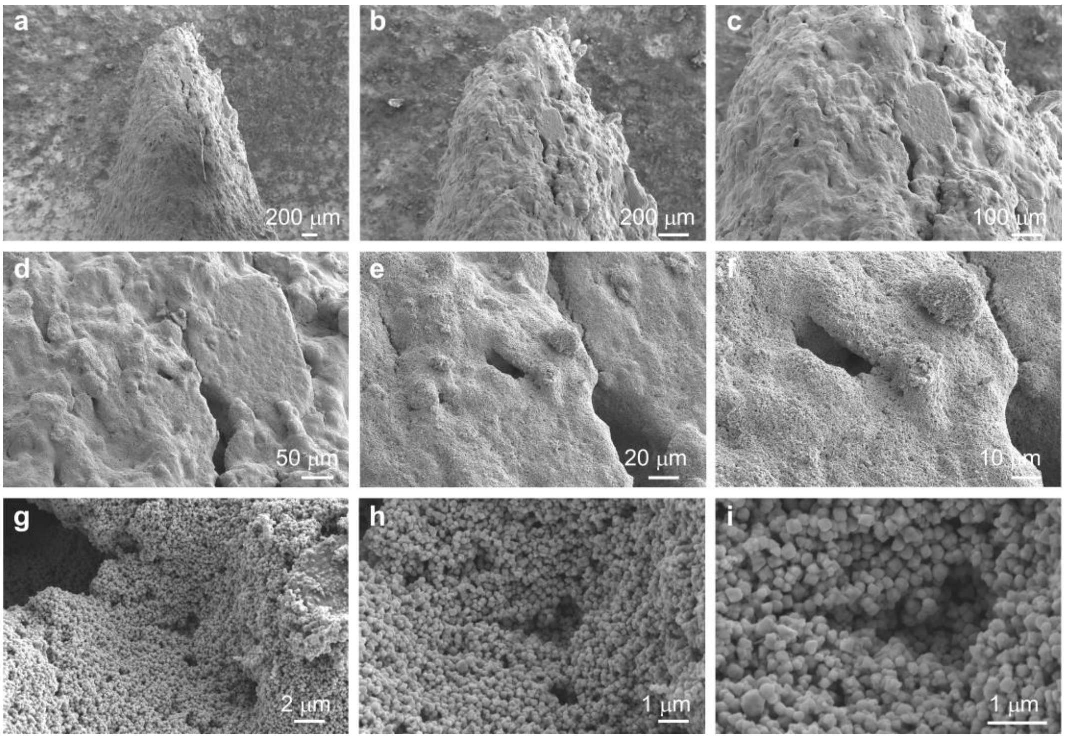

Supplementary Figure 30. SEM images of the vertical conic assembly after dynamic evaporation at different magnified scale.

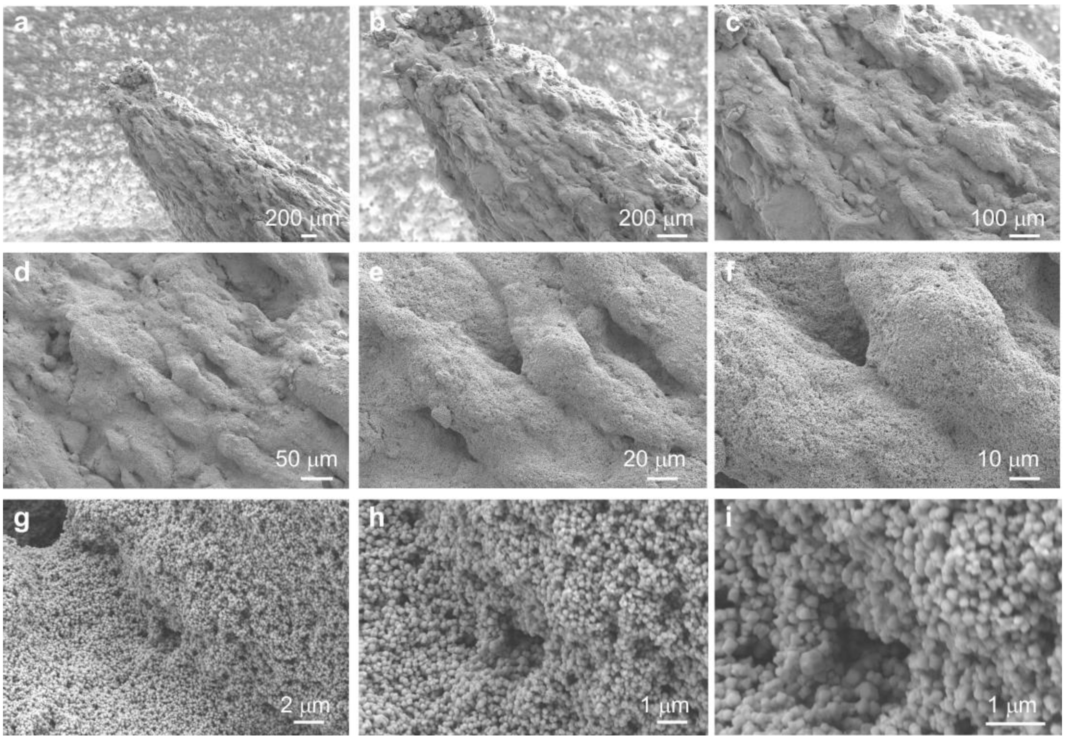

Supplementary Figure 31. SEM images of the left-tilted conic assembly after dynamic

evaporation at different magnified scale.

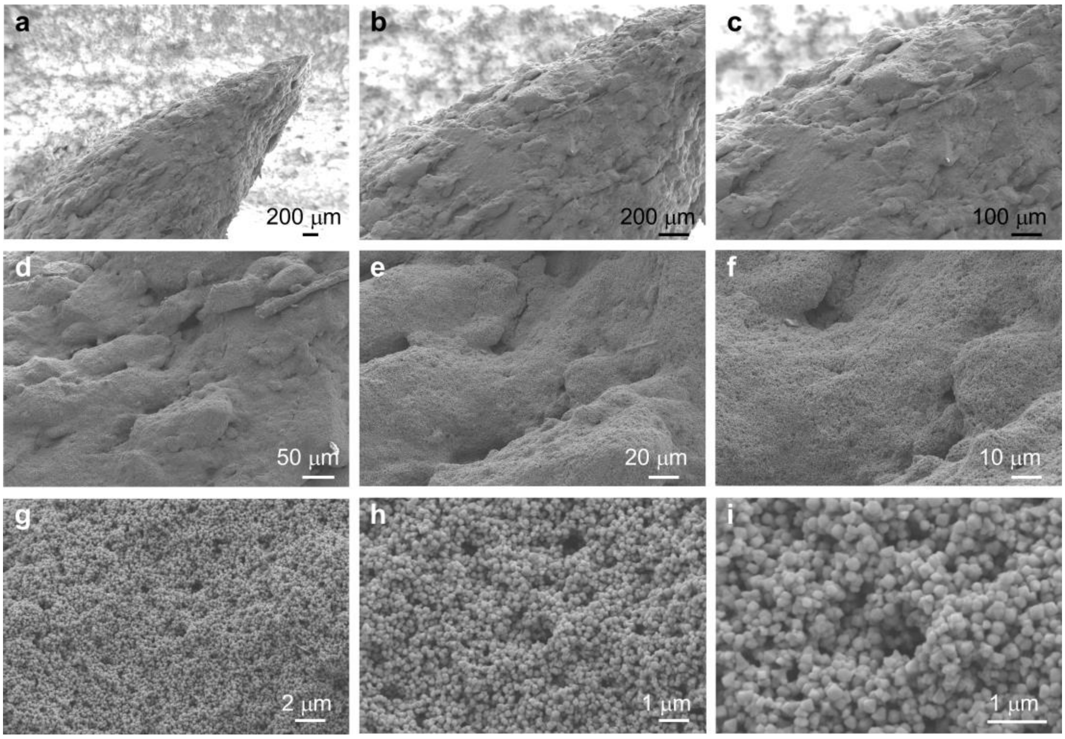

Supplementary Figure 32. SEM images of the right-tilted conic assembly after dynamic evaporation at different magnified scale.

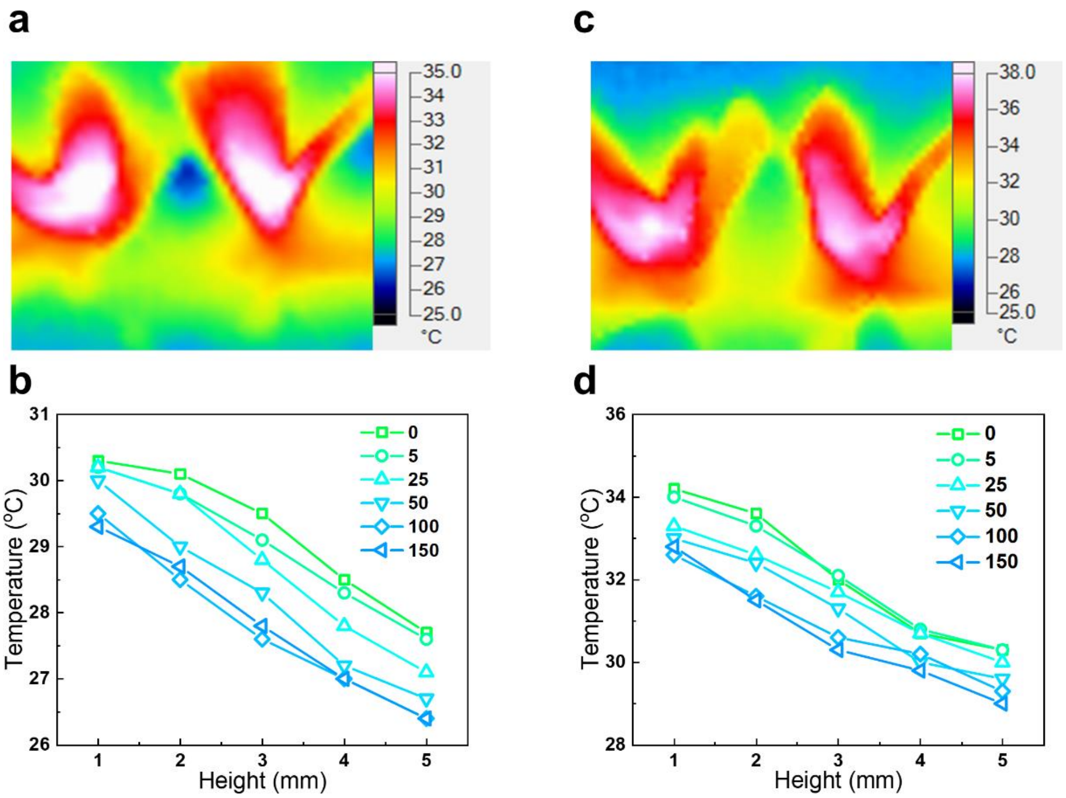

Supplementary Figure 33. Infrared images of dynamic evaporation process of conic

assembly under 1 sun (a, b) and 2 sun illumination (c, d) and the corresponding temperature distribution. With rotation rate increasing, both temperature distributions of 1 sun and 2 sun illumination decrease, and become stable when rotation rate is higher than 100 rpm.

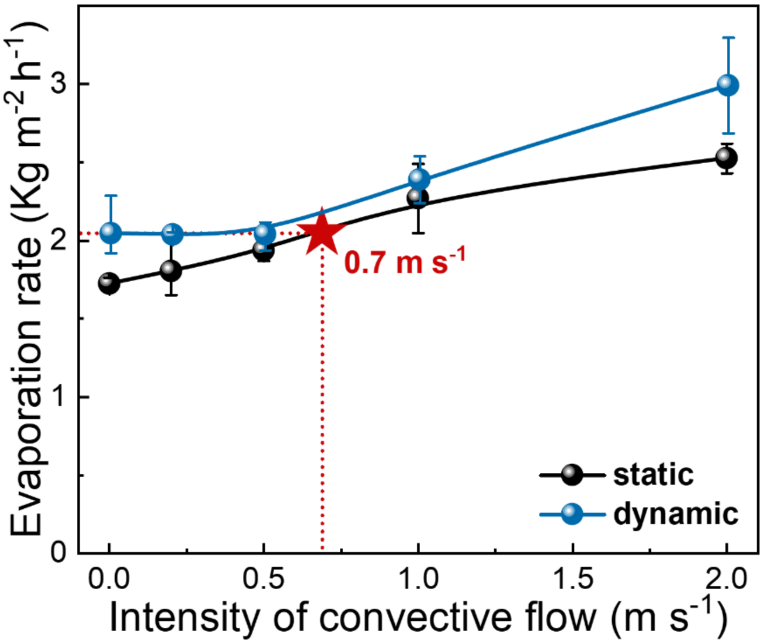

Supplementary Figure 34. Evaporation rate of the CA assembly under convective flow under 1 sun illumination, and 150 rpm rotation rate for dynamic evaporation (n = 3, error bars: standard deviation).

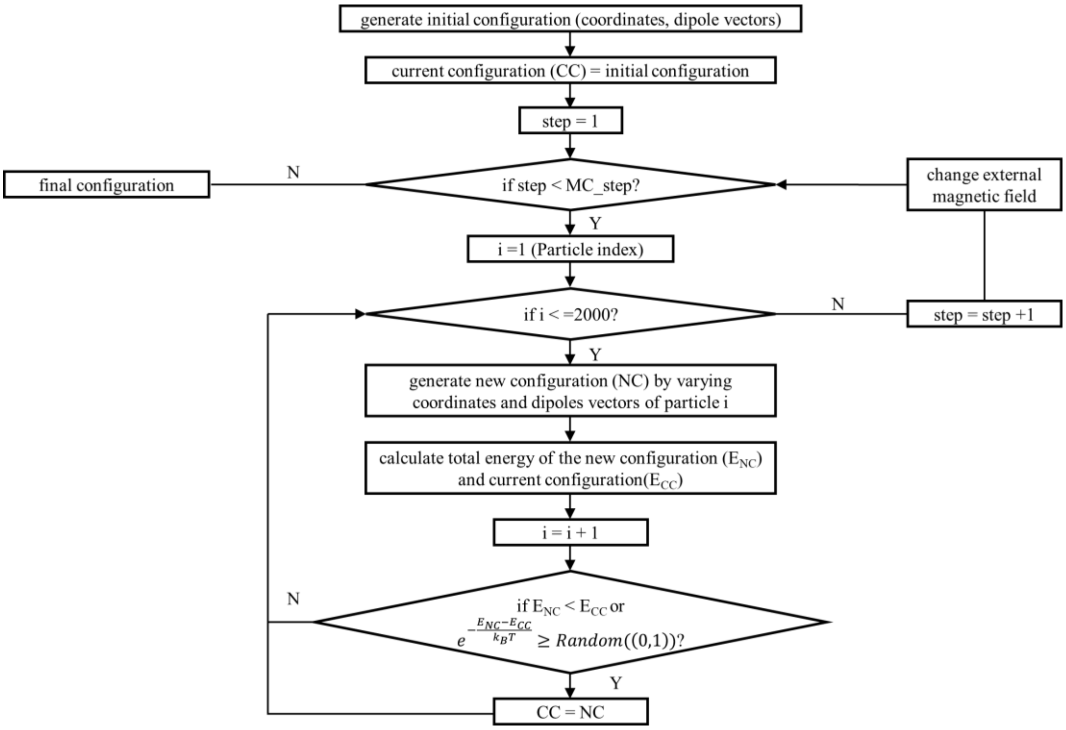

Supplementary Figure 35. The logic diagram of the Monte Carlo simulation.

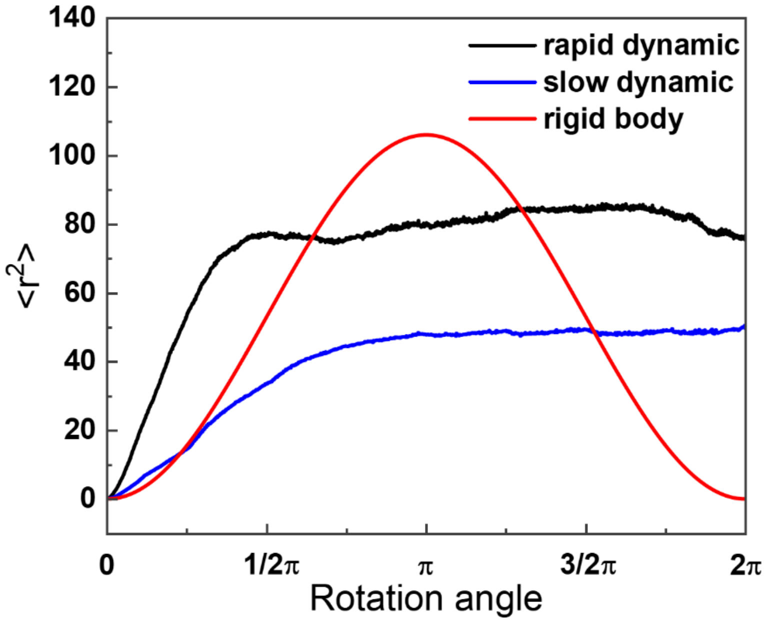

Supplementary Figure 36. Statistical results of fast dynamic, slow dynamic and rigid body rotation process, the value of $<r^2>$ represents the extent of relative movement activity of the particles inside the conic assembly.

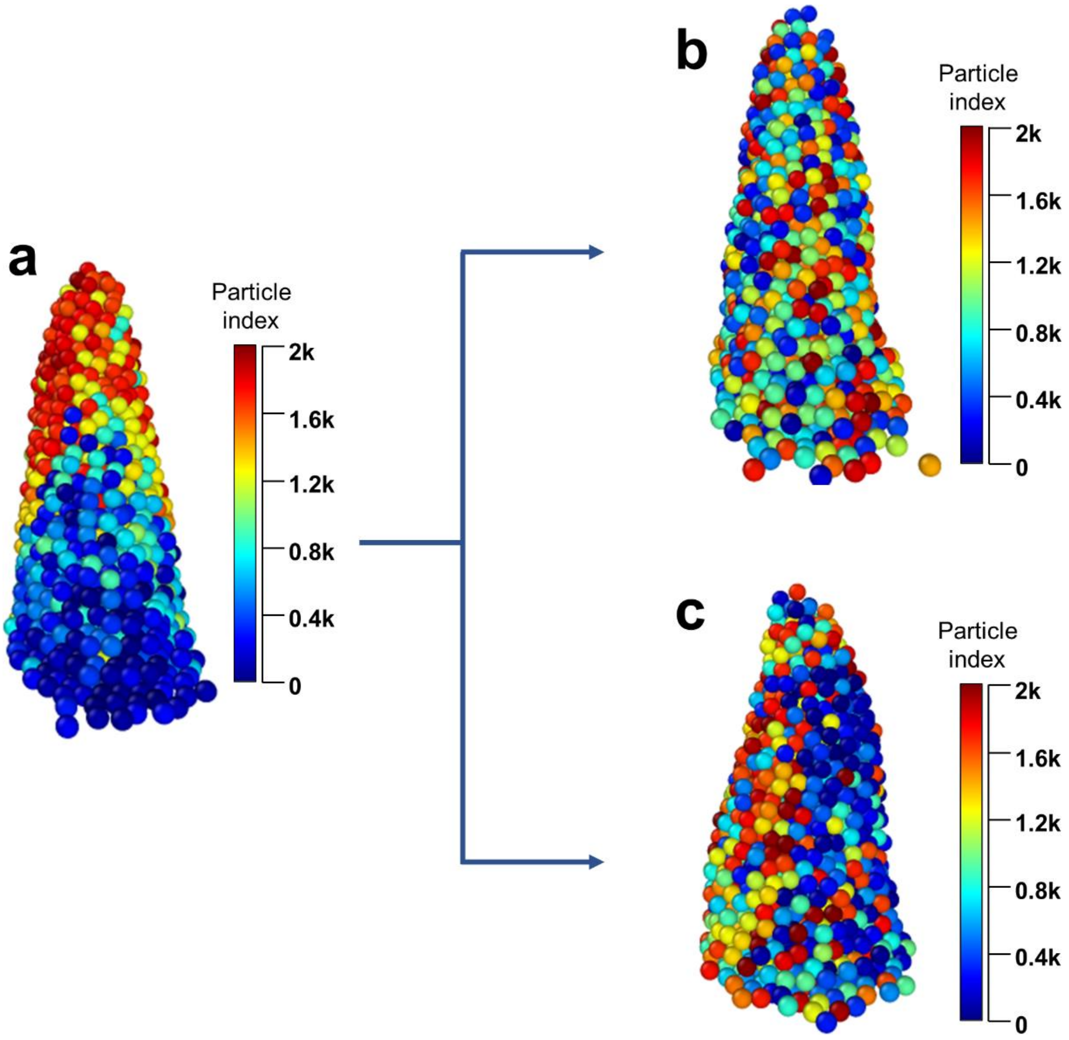

Supplementary Figure 37. The simulated nanoparticles' configuration during dynamic rotation. (a) The initial configuration, (b) configuration of the nanoparticles of the fast dynamic rotation process, and (c) of the slow dynamic rotation process. Particles are colored by the numerical index of the beginning state. From the degree of chaos of the particles, it can be concluded that the stability of the conic shape requires thoroughly reconfiguration of the nanoparticles. However, the slow dynamic rotation process results in severe abnormal deformation of the conic shape.

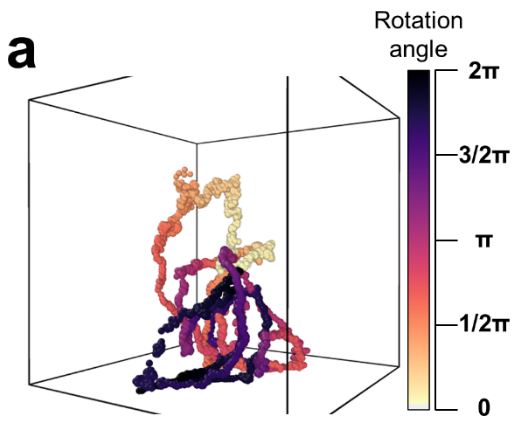

Supplementary Figure 38. Four individual nanoparticles' trajectories during one circular rotation. (a) The fast dynamic rotation, (b) and the slow dynamic rotation. As illustrated, the fast dynamic rotation results in entire reconfiguration of the nanoparticles. However, the nanoparticles movements are restricted in the slow dynamic rotation.

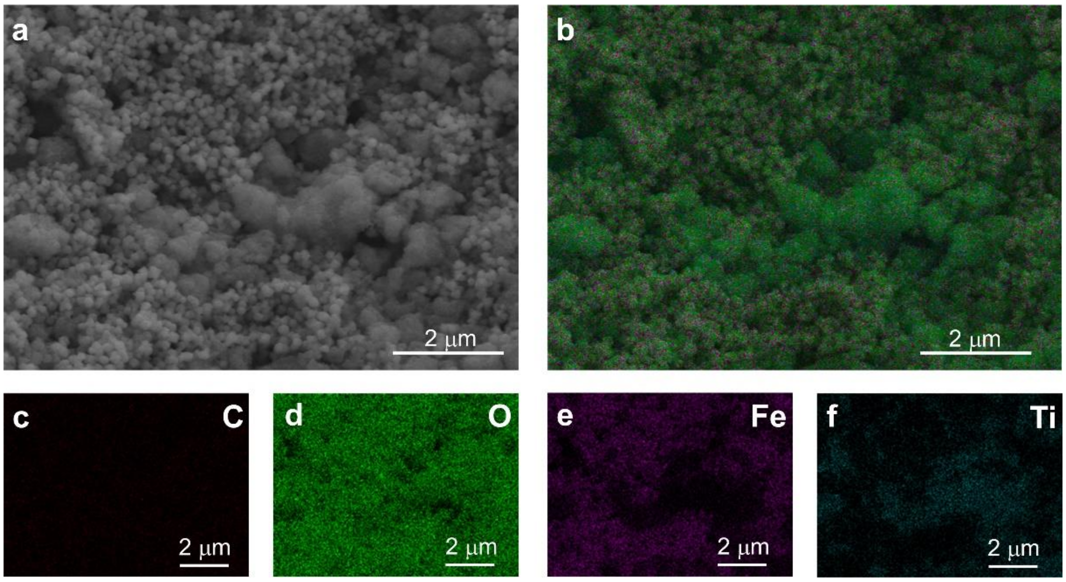

Supplementary Figure 39. EDS mapping of CA assembly containing TiO_2 and Fe_3O_4@G nanoparticles. (a) The original SEM image, (b) the composite image of different elements, EDS mappings of C (c), O (d), Fe (e), and Ti (f) elements. TiO_2 nanoparticles have a similar particle size to Fe_3O_4@G nanoparticles, and could be evenly mixed with Fe_3O_4@G nanoparticles.

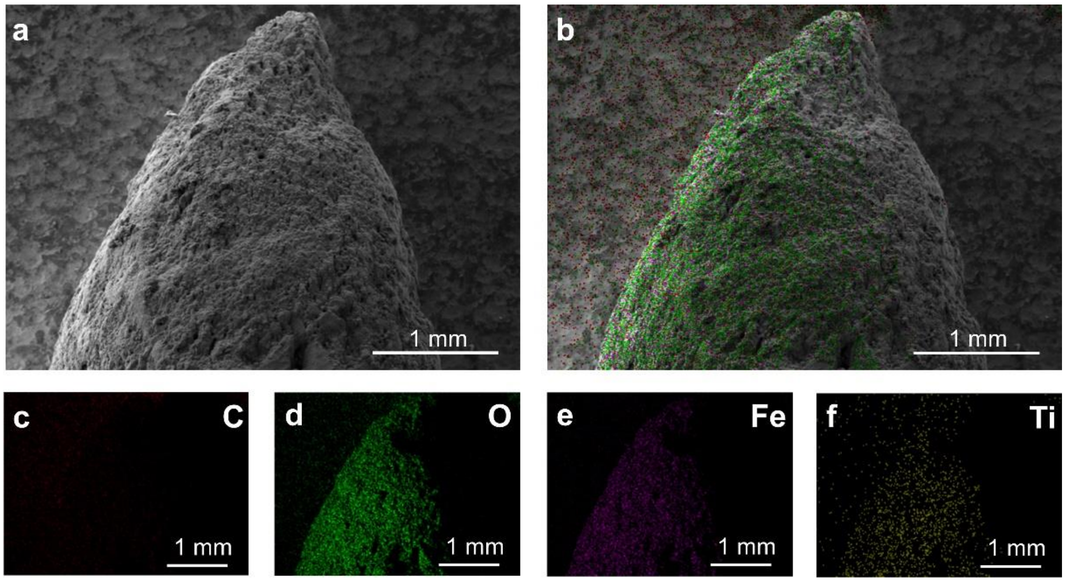

Supplementary Figure 40. EDS mapping of CA assembly containing TiO_2 and Fe_3O_4@G nanoparticles after dynamic rotation. (a) The original SEM image, (b) the composite image of different elements, EDS mappings of C (c), O (d), Fe (e), and Ti (f) elements. After rotation, the TiO_2 nanoparticles redistributed inside the conic assembly from top down, and the distribution was considerably uniform.

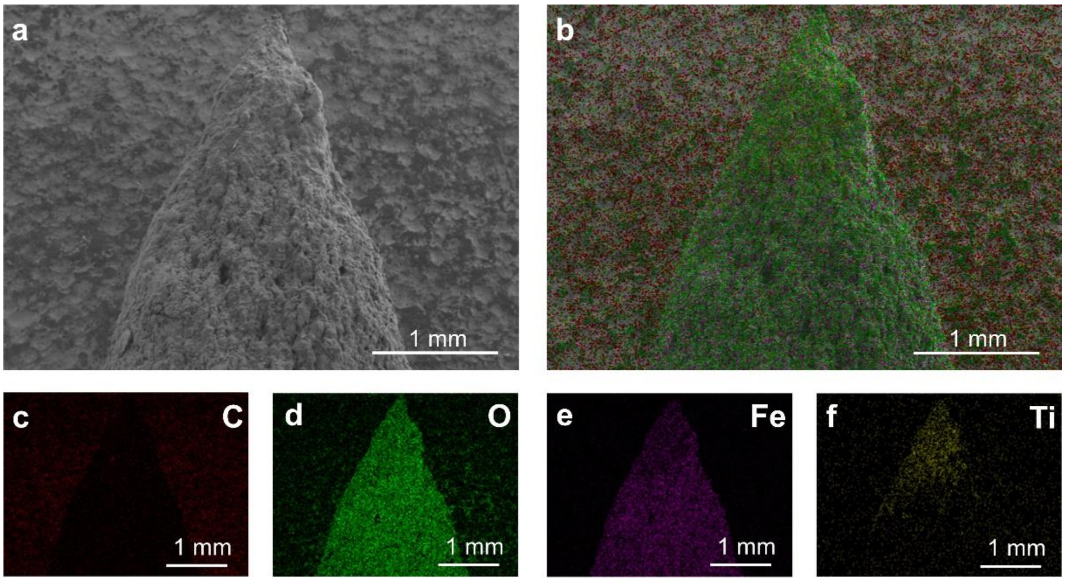

Supplementary Figure 41. EDS mapping of CA assembly containing TiO_2 and Fe_3O_4@G nanoparticles without rotation. (a) The original SEM image, (b) the composite image of different elements, EDS mappings of C (c), O (d), Fe (e), and Ti (f)

elements. TiO_2 nanoparticles were deposited at the top of the conic assembly at the beginning, and no change of the Ti elements' distribution was found if the conic assembly stayed still without rotation.

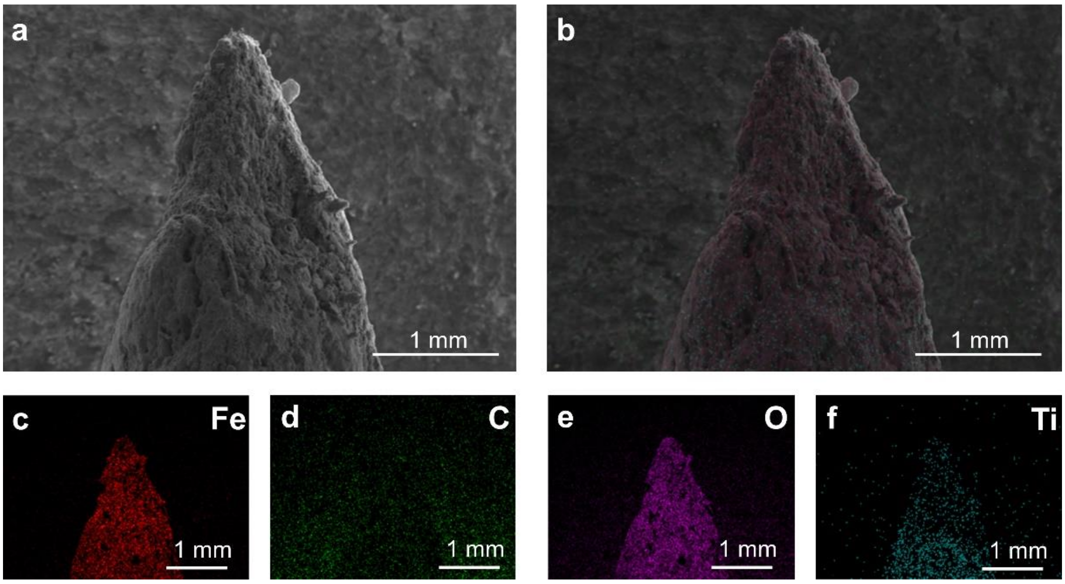

Supplementary Figure 42. EDS mapping of CA assembly containing TiO_2 and Fe_3O_4@G nanoparticles after rotation. (a) The original SEM image, (b) the composite image of different elements, EDS mappings of Fe (c), C (d), O (e), and Ti (f) elements. After rotation, the TiO_2 nanoparticles redistributed inside the conic assembly from bottom up, and the distribution was considerably uniform.

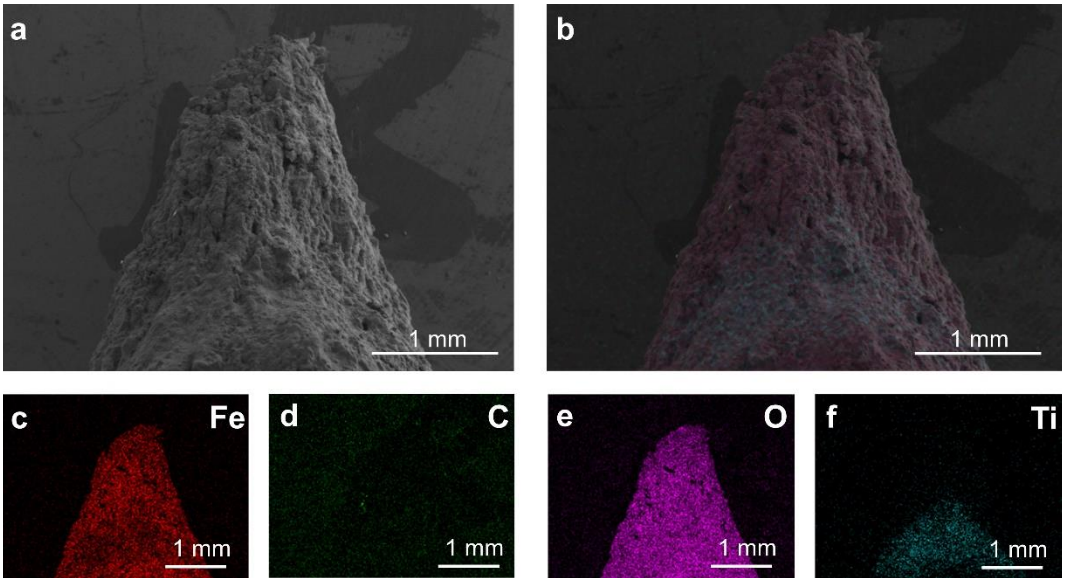

Supplementary Figure 43. EDS mapping of CA assembly containing TiO_2 and Fe_3O_4@G nanoparticles without rotation. (a) The original SEM image, (b) the composite image of different elements, EDS mappings of C (c), O (d), Fe (e), and Ti (f) elements. TiO_2 nanoparticles were deposited at the bottom of the conic assembly at the beginning, and no change of the Ti elements' distribution was found if the conic assembly stayed still without rotation.

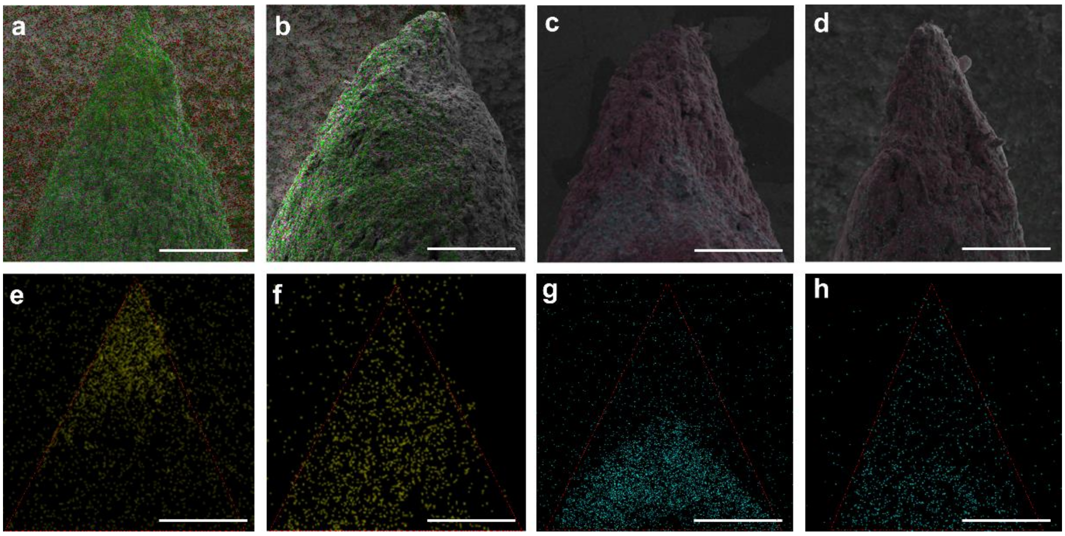

Supplementary Figure 44. TiO_2 nanoparticles' redistribution inside the conic assembly. (a-d) The composite images of the conic assembly, (a) top-deposited conic assembly before rotation, (b) top-deposited conic assembly after rotation, (c) bottom-deposited conic assembly before rotation, and (d) bottom-deposited conic assembly after rotation. (e-h) The EDS mapping of Ti element inside the conic assembly, (e) top-deposited conic assembly before rotation, (f) top-deposited conic assembly after rotation, (g) bottom-deposited conic assembly before rotation, and (h) bottom-deposited conic assembly after rotation. Scale bar, 2 mm.

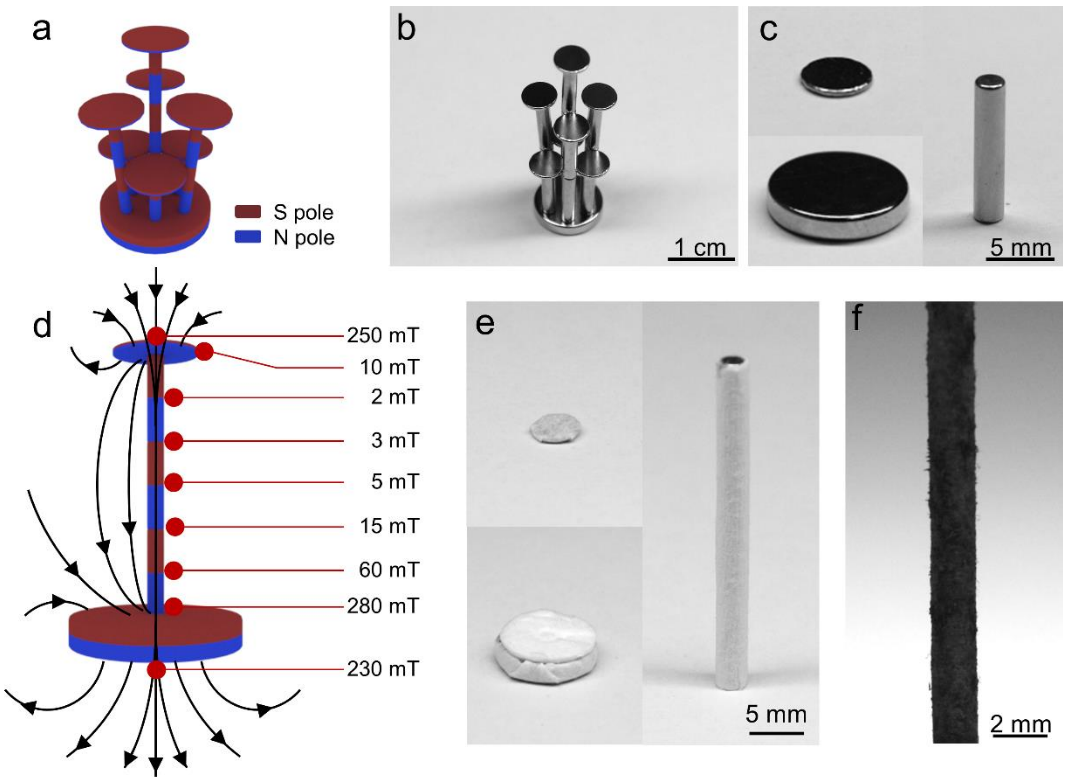

Supplementary Figure 45. Schematic image of the construction of the magnetic hierarchical assembly. (a-c) The schematic image and the optical image of the macroscopic magnets assembly and the component magnets. (d) The schematic image of the magnetic field of the center connection structure, the ligand notes indicate the magnetic field intensity. (e) The optical images of the air-laid paper wrapped macroscopic magnets. (f) The magnified optical image of the stalk showing the distribution state of the Fe_3O_4@G nanoparticles.

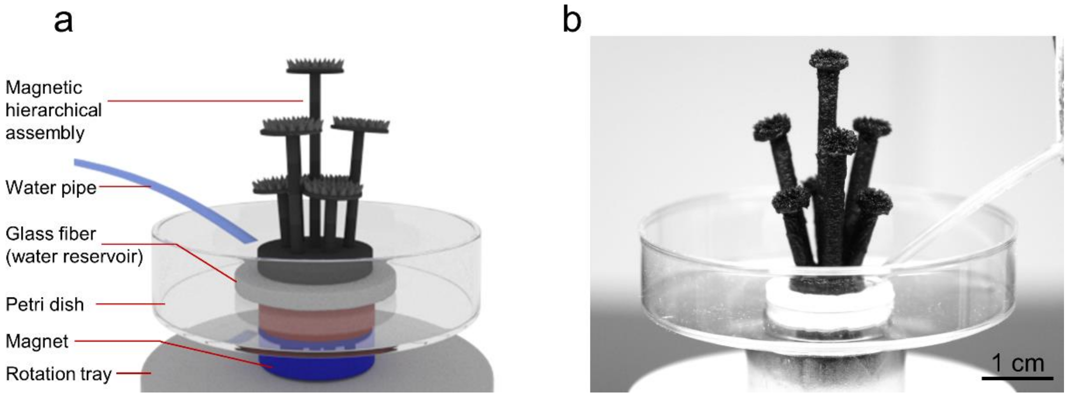

Supplementary Figure 46. Schematic and optical images of the dynamic evaporation

apparatus for magnetic hierarchical assembly. (a, b) The schematic image (a) and optical image (b) of the dynamic apparatus.

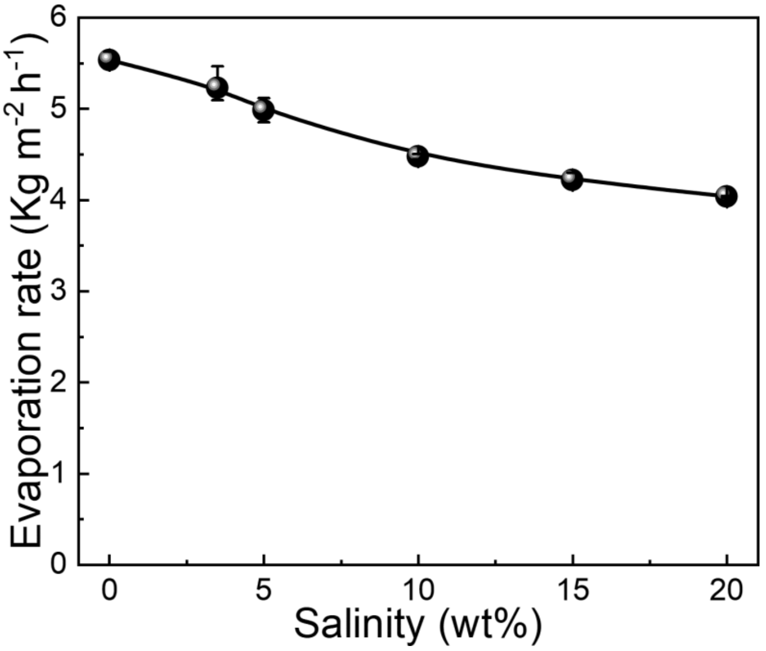

Supplementary Figure 47. Evaporation rates of the magnetic hierarchical assembly with different salinity under 1 sun illumination (n = 3, error bars: standard deviation).

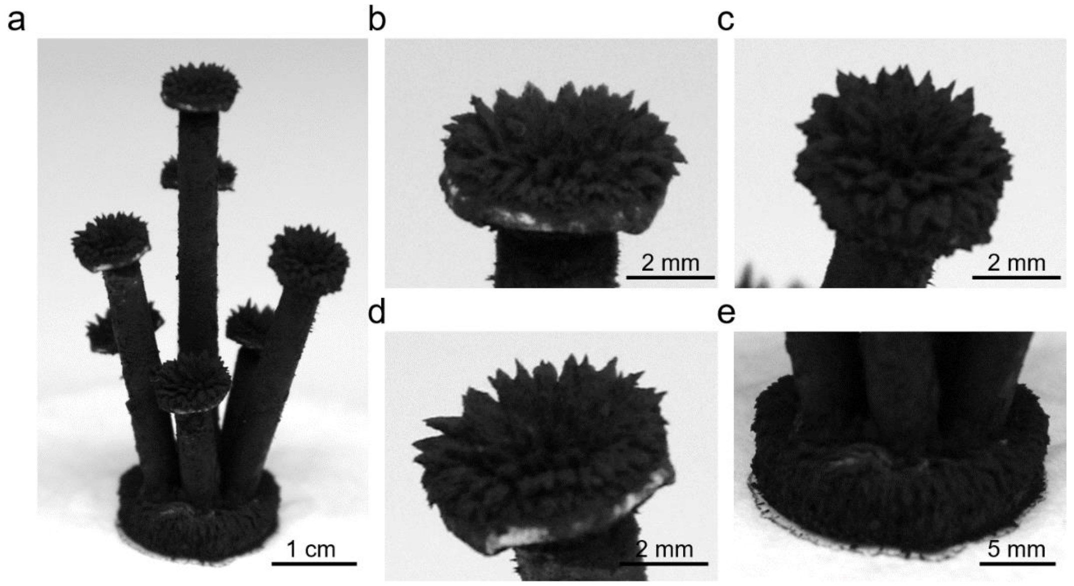

Supplementary Figure 48. Optical images of the magnetic hierarchical assembly after half a year. The full-length photo (a), CA assemblies at the top (b-d), and the CA assembly at the bottom (e).

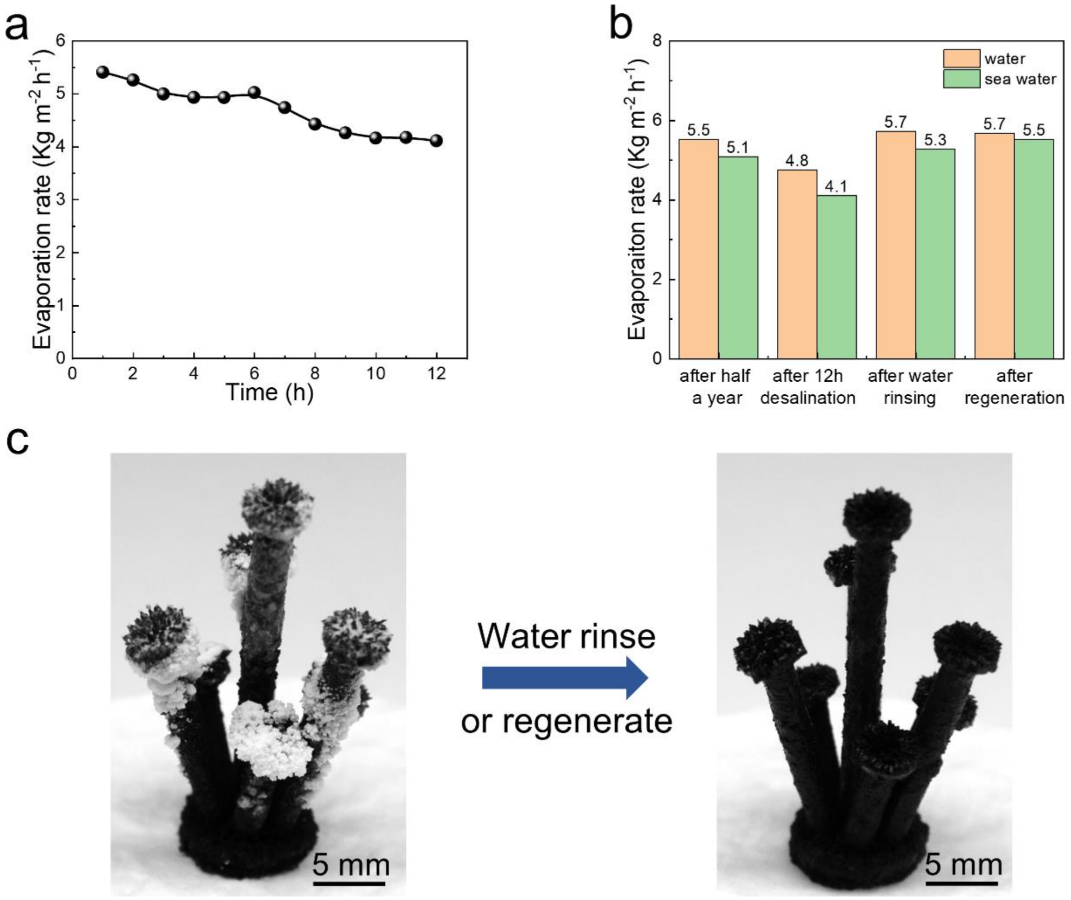

Supplementary Figure 49. The desalination stability of the magnetic hierarchical assembly. (a) 12 hours' desalination test with 3.5 wt% saline under 1 sun illumination. (b) Evaporation rates of the magnetic hierarchical assembly under different circumstances. (c, d) Optical images of the slat contaminated (c) and recovered (d) magnetic hierarchical assembly.

S3. Supplementary Tables

Rotation rate (rpm)		0	25	50	75	100	125	150
Energy input (W m^{-2})	Environmental energy	1572	1782	2014	2088	2122	2173	2190
	Solar-thermal power	1000	1000	1000	1000	1000	1000	1000
Energy consumption(W m^{-2})	Water evaporation	2572	2782	3014	3088	3122	3173	3190

Supplementary Table 1. The energy analysis in the magnetic hierarchical assembly during dynamic evaporation.

Potential	Typical energy	Physical quantity	Scaled value in simulation
Lennard Jones potential	3.37×10^{-19} J	ε	0.003
		σ	1
Magnetic dipole interaction	3.29×10^{-18} J	$\mu_0/4\pi r_{ij}^3$	0.03
Magnetic force	7.26×10^{-17} J	B_z	2
		${B_x}^2 + {B_y}^2$	1
Gravitational potential	2.54×10^{-16} J	mg	0.15
Thermal effect	3.89×10^{-18} J	K_BT	0.0345

Supplementary Table 2. The table of parameters in Monte Carlo simulation.

S4. Supplementary References

1 Narasimhan, T. N. Fourier's heat conduction equation: history, influence, and connections. *Rev. Geophys.* **37**, 151-172 (1999).

2 Dalton, J. Experimental essays, on the constitution of mixed gases; on the force of steam or vapour from water and other liquids in different temperatures, both in a Torricellian vacuum and in air; on evaporation; and on the expansion of gases by heat. *Mem. Lit. Philos. Soc. Manchester.* **5**, 535-602 (1798).

3 Zhang, H., Yuan, Y., Sun, Q., Cao, X. & Sun, L. Steady-state equation of water vapor sorption for CaCl2-based chemical sorbents and its application. *Sci. Rep.*

6, 34115 (2016).

4 Landau, D. P. & Binder, K. *A guide to Monte Carlo simulations in statistical physics*. (Cambridge University Press, 2021).
