# Supplementary material for: A reconfigurable and magnetically responsive assembly for dynamic solar steam generation
Source: Nat Commun. 2022 Jul 27;13:4335. doi: 10.1038/s41467-022-32051-3 (PMC9329472; doi:10.1038/s41467-022-32051-3)
Supplement: Supplementary file 3 — Description of Additional Supplementary Files [file 41467_2022_32051_MOESM3_ESM.pdf]

Description of Additional Supplementary Files

File Name: Supplementary Movie 1
Description: The comparison of disassembly processes between bare Fe_3O_4 assembly and Fe_3O_4@G assembly.

File Name: Supplementary Movie 2
Description: Dynamic rotation of the CA assembly of Fe_3O_4@G.

File Name: Supplementary Movie 3
Description: Temperature distribution of the CA assembly during dynamic evaporation under 1 and 2 sun illumination.

File Name: Supplementary Movie 4
Description: The simulated nanoparticles' movement in CA assembly during dynamic evaporation.

File Name: Supplementary Movie 5
Description: Luminescence tracing of ions movement in CA assembly at static and dynamic state.
